# Association of *MGMT* and *BIN1* genes with Alzheimer's disease risk across sex and *APOE* ε4 status

**DOI:** 10.1002/alz.13550

**Published:** 2023-12-02

**Authors:** Julie Le Borgne, EADB, GR@ACE, Degesco, EADI, GERAD, DemGene, Philippe Amouyel, Ole Andreassen, Ruth Frikke‐Schmidt, Mikko Hiltunen, Martin Ingelsson, Alfredo Ramirez, Giacomina Rossi, Agustin Ruiz, Pascual Sanchez‐Juan, Rebecca Sims, Kristel Sleegers, Magda Tsolaki, Sven J. van der Lee, Julie Williams, Jean‐Charles Lambert, Céline Bellenguez

**Affiliations:** ^1^ Univ. Lille, Inserm, CHU Lille, Institut Pasteur Lille, LabEx DISTALZ ‐ U1167 ‐ RID‐AGE ‐ Facteurs de risque et déterminants moléculaires des maladies liées au vieillissement Lille France; ^2^ NORMENT Centre University of Oslo Oslo Norway; ^3^ Department of Clinical Medicine University of Copenhagen Copenhagen Denmark; ^4^ Department of Clinical Biochemistry Rigshospitalet Copenhagen Denmark; ^5^ Institute of Biomedicine University of Eastern Finland Kuopio Finland; ^6^ Department of Public Health and Caring Sciences/Geriatrics Uppsala University Uppsala Sweden; ^7^ Krembil Brain Institute University Health Network Toronto Ontario Canada; ^8^ Tanz Centre for Research in Neurodegenerative Diseases, Departments of Medicine and Laboratory Medicine & Pathobiology University of Toronto Toronto Ontario Canada; ^9^ Department of Neurodegenerative Diseases and Geriatric Psychiatry University Hospital Bonn Bonn Germany; ^10^ Division of Neurogenetics and Molecular psychiatry Department of Psychiatry and Psychotherapy University of Cologne, Medical Faculty Cologne Germany; ^11^ German Center for Neurodegenerative Diseases (DZNE Bonn) Bonn Germany; ^12^ Glenn Biggs Institute for Alzheimer's & Neurodegenerative Diseases University of Texas Health Sciences Center San Antonio Texas USA; ^13^ Cluster of Excellence on Cellular Stress responses in Aging‐Associated Diseases (CECAD) University of Cologne Cologne Germany; ^14^ Fondazione IRCCS Istituto Neurologico Carlo Besta Milan Italy; ^15^ Research Center and Memory Clinic Fundació ACE Institut Català de Neurociències Aplicades Universitat Internacional de Catalunya Barcelona Spain; ^16^ CiBERNED, Network Center for Biomedical research in Neurodegenerative Diseases National Institute of Health Carlos III Madrid Spain; ^17^ Alzheimer's Centre Reina Sofia‐CIEN Foundation Madrid Spain; ^18^ Division of Psychological Medicine and Clinical Neuroscience, School of Medicine Cardiff University Cardiff UK; ^19^ Complex Genetics of Alzheimer's Disease Group VIB Center for Molecular Neurology, VIB Antwerp Belgium; ^20^ Department of Biomedical Sciences University of Antwerp Antwerp Belgium; ^21^ First Department of Neurology Medical School Aristotle University of Thessaloniki Thessaloniki Greece; ^22^ Alzheimer Hellas Thessaloniki Greece; ^23^ Genomics of Neurodegenerative Diseases and Aging, Human Genetics Vrije Universiteit Amsterdam, Amsterdam UMC location VUmc Amsterdam The Netherlands; ^24^ Alzheimer Center Amsterdam, Neurology Vrije Universiteit Amsterdam, Amsterdam UMC location VUmc Amsterdam The Netherlands; ^25^ Amsterdam Neuroscience Neurodegeneration Amsterdam The Netherlands; ^26^ UKDRI@ Cardiff, School of Medicine Cardiff University Cardiff UK

Chung et al. reported a novel association of the Alzheimer's disease (AD) risk with genetic variants in the *MGMT* gene in women.^1^ The genome‐wide significant signals were found in women lacking the apolipoprotein E ε4 allele (*APOEε4*‐) from 30 studies of the Alzheimer's Disease Genetics Consortium (ADGC) (3399 AD cases and 6905 controls), and in a Hutterite cohort (31 members of a consanguineous kindred with different *APOEε4* statuses, including 5 AD cases who were all women). The effect sizes reported were large: odds ratio [OR] = 1.44 [1.26–1.64], *P* = 4.95 × 10^‐8^ in ADGC for rs12775171, and OR = 2.02 [1.80–2.26], *P* = 1.9 × 10^‐14^ in the Hutterites for rs2803456 and rs12256016. The association found in the ADGC was consistent across studies and not significant in the three other subsets defined by sex and *APOEε4* status (women *APOEε4*+, men *APOEε4*‐, and men *APOEε4*+) for which effect sizes were not reported.

We aimed at replicating the association of *MGMT* with AD risk in the meta‐analysis of 6 case–control studies from the European Alzheimer & Dementia Biobank (EADB) consortium: EADB‐core,^2^ EADI (European Alzheimer's Disease Initiative),^3^, ^4^ GERAD (Genetic and Environmental Risk in AD),^5^ DemGene,^6^ GR@ACE‐DEGESCO,^7^ and Bonn.^2^ We considered a total of 33,677 AD cases and 48,158 controls, all of European ancestry, including 10,354 AD cases and 19,910 controls who were female and *APOEε4*‐ (Figure 1, Tables S1, and S2 in supporting information). The samples were genotyped with different chips and then imputed using[Fig alz13550-fig-0001] the TOPMed reference panel^2^ (supporting information). In each study, we tested the association of *MGMT* variants with AD in the four subsets defined by sex and *APOEε4* status. Analyses were adjusted on principal components, and results were combined across studies in a fixed effect meta‐analysis with an inverse‐variance weighted approach (supporting information).

**FIGURE 1 alz13550-fig-0001:**
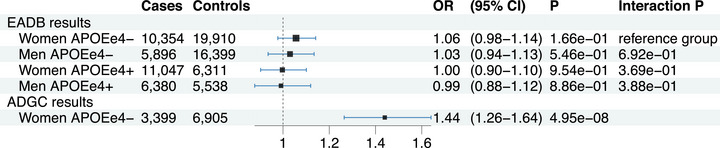
Results of rs12775171 association with Alzheimer's disease (AD) risk in apolipoprotein E (*APOE) ε4*‐ women and the other sex‐*APOEε4* subsets compared with the effect reported in the Alzheimer's Disease Genetics Consortium (ADGC) *APOEε4*‐ women from Chung et al. 2022. The effect allele is G with a frequency of 0.06 in all models. The black square whose size is proportional to the sample size represents the odds ratio (OR) and the blue line the confidence interval (CI). Interaction *P* are *p*‐values of the heterogeneity test between the different group pairs (1 degree of freedom test) using the *APOEε4*‐ women group as a reference for each test (supporting information). EA, Effect allele; *P*, *p*‐value.

None of the *MGMT* variants identified by Chung et al. were found to be associated with AD risk (*P* < 0.05) in the different subsets (Figures S1–S6 in supporting information). The effect of rs12775171 was larger in *APOEε4*‐ women (OR = 1.06 [0.98–1.14], *P* = 0.17) than in the other subsets (OR = 1.03, 1.00, and 0.99 in *APOEε4*‐ men, *APOEε4*+ women, and *APOEε4*+ men, respectively), but those differences were not significant (*P* = 0.69, 0.37, and 0.39 for the comparison of the OR in *APOEε4*‐ women with the one in *APOEε4‐* men, *APOEε4+* women, and *APOEε4*+ men, respectively, Figure 1). Of note, our study in *APOEε4*‐ women had more than 99% power to detect the association with rs12775171 as described by Chung et al., at the nominal significance level of 0.05 (supporting information).

The authors also identified in ADGC *APOEε4‐* women a genome‐wide significant association with AD for a known AD gene, *BIN1* (rs11680911, OR = 1.21 [1.13–1.29], *P* = 2.22 × 10^−8^). We sought to assess whether this association differed across the four sex‐*APOEε4* subsets. We detected a genome‐wide significant association (*P* < 5 × 10^−8^) with AD risk for rs11680911 in *APOEε4*‐ women (OR = 1.12 [1.07–1.16], *P* = 2.21 × 10^−8^) and in *APOEε4*‐ men (OR = 1.16 [1.10–1.21], *P* = 1.75 × 10^−9^), but not in the 2 other subsets (Figures S7–S8 in supporting information). However, the effects in all the subsets were similar (OR = 1.12, 1.16, 1.14, and 1.13 in *APOEε4*‐ women, *APOEε4*‐ men, *APOEε4*+ women, and *APOEε4*+ men, respectively), and effects were not significantly different between the subsets (Figures S7–S8 and Table S3 in supporting information).

We performed several sensitivity analyses in the EADB studies (supporting information, Tables S1–S4 and Figures S1–S12), but none of them identified a significant association of *MGMT* with AD risk in *APOEε4*‐ women or differences of association between subsets in *BIN1*.

In conclusion, we did not find a significant, nor suggestive association of the *MGMT* variants identified by Chung et al. with AD risk, in any of the subsets defined by sex and *APOEε4* status, where our sample size was up to three times larger than in the original publication. Additionally, we did not identify a significant effect difference of *BIN1* rs11680911 variant across sex and *APOEε4* status subsets.

## CONFLICT OF INTEREST STATEMENT

Martin Ingelsson is a paid consultant to BioArtic.

## FUNDING INFORMATION

This study was supported by a grant from the Fondation pour la Recherche sur Alzheimer, convention 2022‐A‐01, the JPco‐fuND‐2 “Multinational research projects on Personalized Medicine for Neurodegenerative Diseases” PREADAPT project (ANR‐19‐JPW2‐0004), and the JPco‐fuND EADB grant. Ole Andreassen was supported by the Research Council of Norway (RCN grants 223273, 283799, 324252, 344121). Alfredo Ramirez was supported by the German Federal Ministry of Education and Research (BMBF: 01ED1619A). Agustin Ruiz was supported by GRIFOLS‐GR@ACE DEGESCO, LA CAIXA‐GR@ACE DEGESCO and ISCIII‐Ministry of Health Spain. Rebecca Sims was supported by the Medical Research Council UK. Julie Williams was supported by UKDRI‐IPSC Platform to Model Alzheimer's Disease Risk (IPMAR).

## Supporting information

Supporting Information

Supplemental Table 1‐4

Supporting Information
