# Supplementary material for: Association of MGMT and BIN1 genes with Alzheimer's disease risk across sex and APOE ε4 status
Source: Alzheimers Dement. 2023 Dec 2;20(3):2282–4. doi: 10.1002/alz.13550 (PMC10984453; doi:10.1002/alz.13550)
Supplement: Supplementary file 1 — Supporting Information [file ALZ-20-2282-s001.pdf]

Supporting information

Table of content:

1) Samples

2) Association tests

3) Sensitivity analyses

4) Power study

5) Supplementary References

6) Supplementary Tables

7) Supplementary Figures

8) Consortia author lists

1) Samples

The samples, and the quality control and imputation protocols are described in Bellenguez et al. [1]. All samples were imputed with the TOPMed imputation panel.

Sex was defined as the self-reported sex, or, when missing, as the genetically determined sex. Samples with discordant sex between self-reported and genetically determined sex were excluded.

Age of AD cases is defined as the age at onset, if available. Otherwise, we used, by order of priority, the baseline age, the age at last exam and the age at death. For controls, age is defined as the age at last exam, and if not available the age at death and the baseline age, by order of priority.

APOEε4 status is a binary variable defined by the presence or absence of at least one *APOE* ε4 allele, which was determined from the genotyped *APOE* status specified in the clinical file of the study (ε4ε4, ε2ε4 and ε3ε4 samples are *APOE*ε4+ and all others *APOE*ε4-). If unavailable in the clinical file of the study, *APOEε4* status was defined using the imputed data; rs429358 and rs7412, the two *APOE* variants, had a good imputation quality (r2 > 0.8) in all studies. For a given individual, genotypes of the two *APOE* variants were only considered if their probability was higher than 0.8. This means that *APOE* status could be missing even after imputation. For samples with both genotyped and imputed *APOE* status available, the *APOE* status was set to missing if the genotyped and imputed statuses were different.

In a sensitivity analysis, we additionally excluded individuals who only had an imputed *APOE* status.

2) Association tests

For the stratified models, we performed logistic regressions of the AD status on the genetic variants with an additive genetic model using SNPTEST "newml" method [2]. Analyses were adjusted on principal components and other study-specific variables, when necessary, such as the genotyping center for EADB-core and the genotyping chip for Bonn. Each stratified model included only samples from one of the subsets defined by sex and *APOEε4* status (women *APOEε4*-, men *APOEε4*-, women *APOEε4*+ and men *APOEε4*+).

A quality control of the results was carried out for all datasets. We filtered out variants with at least one missing datum (on effect, standard error, or p-value), an absolute effect size greater than 5, or an imputation quality less than 0.3. We also filtered out the variants whose expected minimum effective allele count across cases and controls was less than 5. Effective allele count is defined as the product of the imputation quality and the expected minor allele count (2 x MAF x sample size) [3].

For each subset, we computed an effect size across studies (β) and its corresponding standard error (se) by performing a fixed effect meta-analysis with an inverse-variance weighted approach as implemented in METAL [4].

After the meta-analysis, we filtered the variants analyzed in less than 40% of the AD cases, variants with too much heterogeneity between the studies (p-value of heterogeneity less than $5x10^{-8}$) and variants with too large frequency amplitude between the studies (difference between the minimum frequency and the maximum frequency above 0.4).

To compare the variant effects across two subsets, we performed the test for heterogeneity between two groups (1 degree of freedom test) defined by Magi et al. [5], which is equivalent to an interaction test. For each interaction test, we used the women *APOEε4*- group as reference. In practice, we computed the interaction p-value with a Wald test using the effect size (β_I) and corresponding standard error (se_I) of the interaction between two groups:

$$\beta_I = \beta_O - \beta_R ; se_I = \sqrt{se_O^2 + se_R^2}$$

Where β_R and β_O are the effect sizes of the reference group and the group of interest, respectively, and se_R and se_O are their standard errors.

Additionally, we compared all pairs of sex-*APOEε4* subsets for *BIN1* variants (table S3).

3) Sensitivity analyses

a. Age and *APOEε4* status sensitivity analysis

We first performed age-adjusted sensitivity analyses in the EADB studies where age is not too imbalanced between cases and controls. EADI, EADB-core, Bonn and DemGene studies were included, for a total of 23,727 AD cases and 29,484 controls, including 6,704 AD cases and 13,030 controls among women *APOEε4*- (table S1).

Neu et al. [6] found an interaction between sex and *APOE* status for AD in younger ages only, i.e., individuals between the age of 65 and 75 years. We thus performed a sensitivity analysis to younger ages by including only individuals within this age range, which represents 1,926 AD cases and 4,380 controls among women *APOEε4*- (table S1). All EADB studies were included in this analysis.

We also performed an *APOEε4* status sensitivity analysis by excluding individuals who only had an imputed *APOE* status (EADI, EADB-core, Bonn and GERAD studies were included). We thus considered a total of 18,351 AD cases and 25,795 controls, including 5,519 AD cases and 11,237 controls among women *APOEε4*- (table S1).

Results were consistent across the main analysis and those three sensitivity analyses for both *MGMT* and *BIN1* variants (figures S1, S3, S5 and S7).

b. Whole locus analysis

We extended the analysis in women *APOEε4*- to the whole *MGMT* and *BIN1* loci.

The variant with the lowest p-value in *MGMT* was rs138350418 with minor allele frequency (MAF) = 1%, OR = 1.26 [1.08-1.46], P = 2.7×10^{-3}, effect allele (EA) = A, which is not significant after Bonferroni correction for 8209 independent tests (figures S9-S10). To compute the number of independent tests, we defined independent variants among all variants tested with the PLINK pruning procedure; in each step, pairs of variants are considered, and one variant is excluded from each variant pair with a r^2 higher than 0.2 and located within 500kb from each other, until no such pair remains [7]. Of note, rs138350418 is not in linkage disequilibrium (LD, $r^2 < 0.2$) with any of the *MGMT* variants reported by Chung et al.

The variant with the lowest p-value in the BIN1 locus in EADB women *APOEε4*- was rs6733839 with OR = 1.19[1.15-1.24], P = 7.15×10^{-21} (figures S11-S12), which is also the top variant from the genome-wide significant signal in *BIN1* in the association study including all EADB samples [1].

4) Power study

The power to detect the *MGMT* rs12775171 association with AD in women *APOEε4*- was computed using the Sham and Purcell [8] method. We considered a joint analysis of the 30,264 women *APOEε4*- (including 10,354 AD cases) from the six EADB studies using a log-additive coding. We used the rs12775171's minor allele frequency (0.06) and OR (1.44) provided by Chung et al. [9] in women *APOEε4*- and a disease prevalence of 0.05. Our study in women *APOEε4*- had more than 99% power to detect a variant with an OR of 1.44, at the nominal significance level of 0.05.

5) Supplementary References

[1] C. Bellenguez, F. Küçükali, I. E. Jansen, L. Kleineidam, S. Moreno-Grau, N. Amin et al. New insights into the genetic etiology of Alzheimer's disease and related dementias. Nat. Genet. 2022;54(4):412–36.

[2] J. Marchini, B. Howie, S. Myers, G. McVean, P. Donnelly. A new multipoint method for genome-wide association studies by imputation of genotypes. *Nat Genet*. 2007;39(7):906-913. doi:10.1038/ng2088

[3] C.L. Satizabal, H. H. Adams, D. P. Hibar, et al. Genetic architecture of subcortical brain structures in 38,851 individuals. *Nat Genet*. 2019;51(11):1624-1636. doi:10.1038/s41588-019-0511-y

[4] C. J. Willer, Y. Li, G. R. Abecasis. METAL: Fast and efficient meta-analysis of genomewide association scans. *Bioinformatics.* 2010;*26*(17): 2190–2191.doi:10.1093/bioinformatics/btq340

[5] R. Mägi, C. M. Lindgren, A. P. Morris. Meta-analysis of sex-specific genome-wide association studies. *Genetic Epidemiology. 2010;34*(8):846–853.doi:10.1002/gepi.20540

[6] S. C. Neu, J. Pa, W. Kukull, et al. Apolipoprotein E Genotype and Sex Risk Factors for Alzheimer Disease: A Meta-analysis. *JAMA Neurol*. 2017;74(10):1178-1189. doi:10.1001/jamaneurol.2017.2188

[7] S. Purcell, B. Neale, K. Todd-Brown, et al. PLINK: a tool set for whole-genome association and population-based linkage analyses. *Am J Hum Genet*. 2007;81(3):559-575. doi:10.1086/519795

[8] P. C. Sham, S. M. Purcell. Statistical power and significance testing in large-scale genetic studies. *Nature Reviews Genetics. 2014;15*(5):335–346.doi:10.1038/nrg3706

[9] J. Chung, A. Das, X. Sun, et al. Genome-wide association and multi-omics studies identify MGMT as a novel risk gene for Alzheimer's disease among women. Alzheimer's Dement. 2022;10.1002/alz.12719.doi:10.1002/alz.12719:

6) Supplementary Tables

Table S1: Sample size of the EADB meta-analysis in the main analysis and the three sensitivity analyses.

Table S2: Characteristics of the samples in the sex and *APOEε4* status subsets in the EADB studies.

Table S3: P values for the comparison of BIN1 variants effect on AD risk in the different sex-APOEε4 subsets.

Table S4: List of variants studied.

7) Supplementary Figures

rs12775171 (chr10:129604777:T:G), EA = G

	Cases	Controls	OR	(95% CI)	P	Interaction P
EADB results						
Women APOE4-	10,354	19,910	1.06	(0.98-1.14)	1.66e-01	reference group
Men APOE4-	5,896	16,399	1.03	(0.94-1.13)	5.46e-01	6.92e-01
Women APOE4+	11,047	6,311	1.00	(0.90-1.10)	9.54e-01	3.69e-01
Men APOE4+	6,380	5,538	0.99	(0.88-1.12)	8.86e-01	3.88e-01
EADB results (age-adjusted)						
Women APOE4-	6,704	13,030	1.05	(0.95-1.16)	3.48e-01	reference group
Men APOE4-	4,160	9,464	1.06	(0.94-1.20)	3.10e-01	8.60e-01
Women APOE4+	8,007	3,898	1.04	(0.92-1.18)	5.00e-01	9.60e-01
Men APOE4+	4,856	3,092	1.02	(0.88-1.19)	7.58e-01	7.96e-01
EADB results (no imputed APOE status)						
Women APOE4-	5,519	11,237	1.09	(0.98-1.21)	1.09e-01	reference group
Men APOE4-	3,274	8,510	1.09	(0.95-1.24)	2.10e-01	9.96e-01
Women APOE4+	6,031	3,344	1.03	(0.90-1.19)	6.26e-01	5.57e-01
Men APOE4+	3,527	2,704	1.07	(0.90-1.27)	4.24e-01	8.78e-01
EADB results (only 65 to 75 yo)						
Women APOE4-	1,839	4,082	0.97	(0.81-1.16)	7.24e-01	reference group
Men APOE4-	1,418	3,268	0.99	(0.81-1.20)	8.86e-01	8.96e-01
Women APOE4+	3,553	1,445	1.07	(0.88-1.29)	5.03e-01	4.68e-01
Men APOE4+	1,969	1,054	0.97	(0.76-1.22)	7.72e-01	9.90e-01
ADGC results						
Women APOE4-	3,399	6,905	1.44	(1.26-1.64)	4.95e-08	

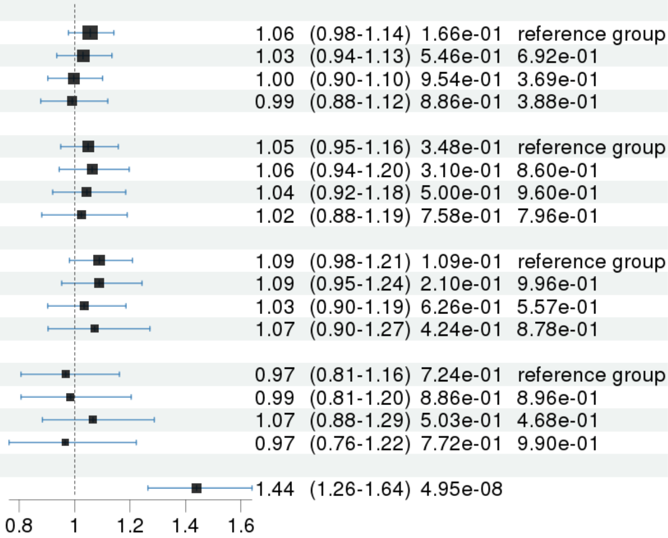

Figure S1: Result of rs12775171 (*MGMT*) in the four sex-*APOEε4* subsets compared with the effect reported in ADGC women *APOEε4*- and the effects found in the age-adjusted, *APOEε4* status and younger ages sensitivity analyses. EA: Effect allele, OR: odds ratio, CI: confidence interval, P: p-value, yo: years old.

a)

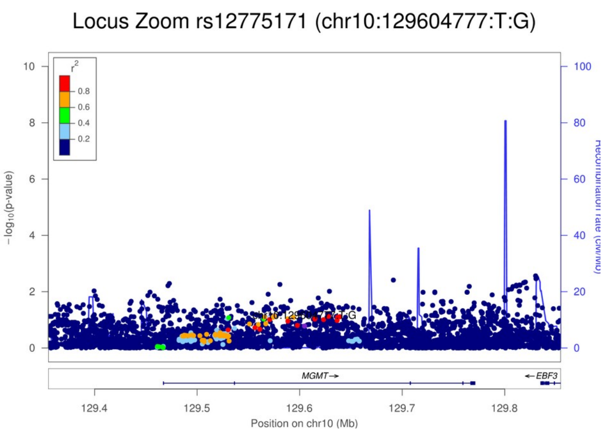
Locus Zoom rs12775171 (chr10:129604777:T:G)
−log10(p-value)
Recombination rate (cM/Mb)
r2
0.8
0.6
0.4
0.2
MGMT→
←EBF3
129.4
129.5
129.6
129.7
129.8
Position on chr10 (Mb)

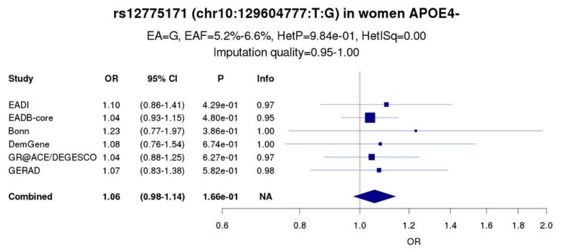
rs12775171 (chr10:129604777:T:G) in women APOE4-
EA=G, EAF=5.2%-6.6%, HetP=9.84e-01, HetISq=0.00
Imputation quality=0.95-1.00
Study OR 95% CI P Info
EADI 1.10 (0.86-1.41) 4.29e-01 0.97
EADB-core 1.04 (0.93-1.15) 4.80e-01 0.95
Bonn 1.23 (0.77-1.97) 3.86e-01 1.00
DemGene 1.08 (0.76-1.54) 6.74e-01 1.00
GR@ACE/DEGESCO 1.04 (0.88-1.25) 6.27e-01 0.97
GERAD 1.07 (0.83-1.38) 5.82e-01 0.98
Combined 1.06 (0.98-1.14) 1.66e-01 NA
OR

b)

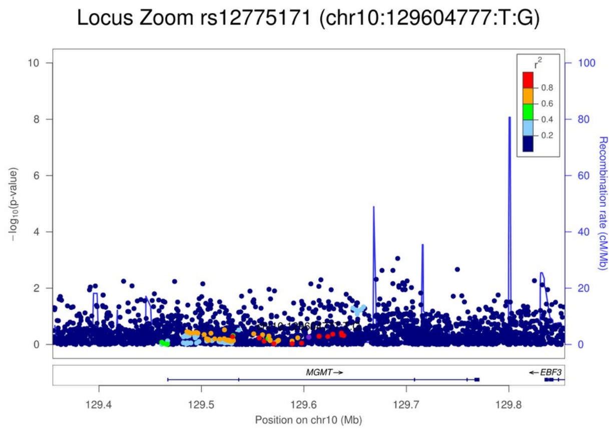
Locus Zoom rs12775171 (chr10:129604777:T:G)
−log10(p-value)
Recombination rate (cM/Mb)
MGMT→
←EBF3
Position on chr10 (Mb)

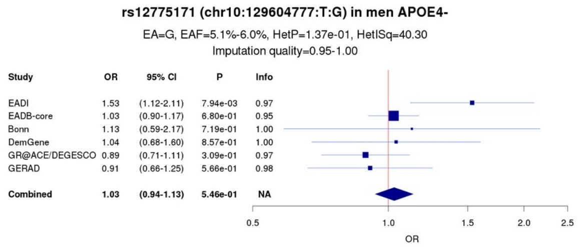
rs12775171 (chr10:129604777:T:G) in men APOE4-
EA=G, EAF=5.1%-6.0%, HetP=1.37e-01, HetISq=40.30
Imputation quality=0.95-1.00
Study OR 95% CI P Info
EADI 1.53 (1.12-2.11) 7.94e-03 0.97
EADB-core 1.03 (0.90-1.17) 6.80e-01 0.95
Bonn 1.13 (0.59-2.17) 7.19e-01 1.00
DemGene 1.04 (0.68-1.60) 8.57e-01 1.00
GR@ACE/DEGESCO 0.89 (0.71-1.11) 3.09e-01 0.97
GERAD 0.91 (0.66-1.25) 5.66e-01 0.98
Combined 1.03 (0.94-1.13) 5.46e-01 NA
OR

c)

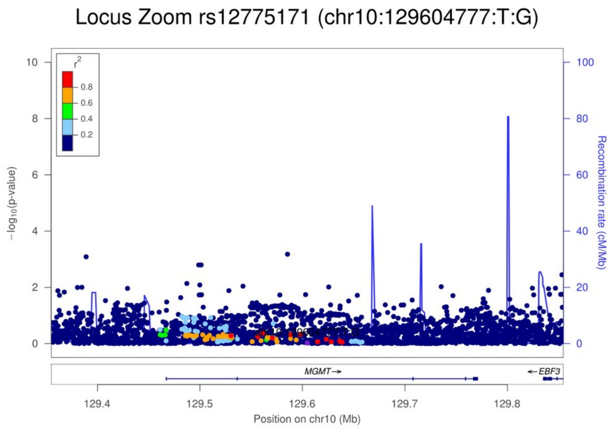
Locus Zoom rs12775171 (chr10:129604777:T:G)
−log10(p-value)
Recombination rate (cM/Mb)
MGMT→
←EBF3
Position on chr10 (Mb)

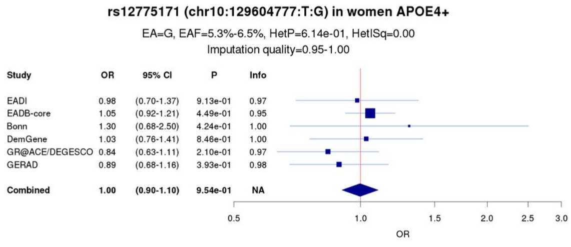
rs12775171 (chr10:129604777:T:G) in women APOE4+
EA=G, EAF=5.3%-6.5%, HetP=6.14e-01, HetISq=0.00
Imputation quality=0.95-1.00
Study OR 95% CI P Info
EADI 0.98 (0.70-1.37) 9.13e-01 0.97
EADB-core 1.05 (0.92-1.21) 4.49e-01 0.95
Bonn 1.30 (0.68-2.50) 4.24e-01 1.00
DemGene 1.03 (0.76-1.41) 8.46e-01 1.00
GR@ACE/DEGESCO 0.84 (0.63-1.11) 2.10e-01 0.97
GERAD 0.89 (0.68-1.16) 3.93e-01 0.98
Combined 1.00 (0.90-1.10) 9.54e-01 NA
OR

d)

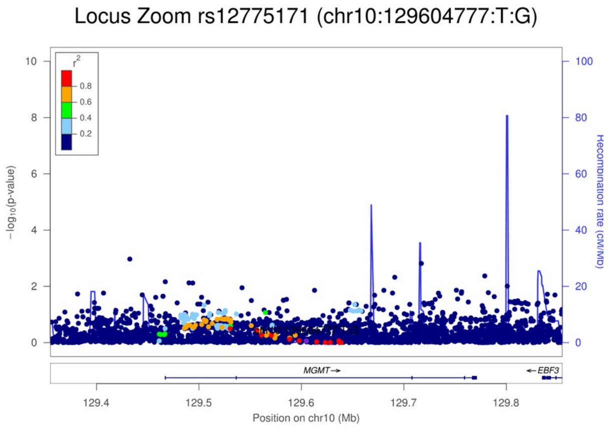

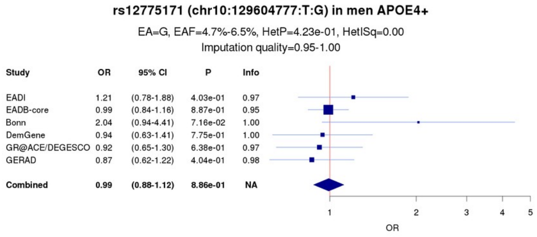

Figure S2: Locus zoom and forest plots of rs12775171 (*MGMT*) in a) women *APOEε4*-, b) men *APOEε4*-, c) women *APOEε4+* and d) men *APOEε4*-. Each locus zoom is based on the meta-analysis of the six EADB studies results in one subset. The variant in purple is rs12775171. The positions are in GRCh38 Assembly. OR: odds ratio, CI: confidence interval, EA: effect allele, EAF: effect allele frequency range across all studies, HetP: heterogeneity P value, HetISq: heterogeneity statistic, info: imputation quality.

rs2803456 (chr10:129266398:C:G), EA = C

	Cases	Controls	OR	(95% CI)	P	Interaction P
EADB results						
Women APOE4-	10,354	19,910	0.98	(0.92-1.04)	4.50e-01	reference group
Men APOE4-	5,896	16,399	0.95	(0.88-1.02)	1.71e-01	5.41e-01
Women APOE4+	11,047	6,311	1.06	(0.98-1.14)	1.61e-01	1.16e-01
Men APOE4+	6,380	5,538	1.02	(0.93-1.12)	6.60e-01	4.40e-01
EADB results (age-adjusted)						
Women APOE4-	6,704	13,030	1.03	(0.95-1.11)	4.91e-01	reference group
Men APOE4-	4,160	9,464	0.94	(0.86-1.03)	1.83e-01	1.42e-01
Women APOE4+	8,007	3,898	1.08	(0.97-1.19)	1.49e-01	4.66e-01
Men APOE4+	4,856	3,092	1.02	(0.91-1.15)	6.83e-01	9.74e-01
EADB results (no imputed APOE status)						
Women APOE4-	5,519	11,237	0.99	(0.92-1.08)	8.96e-01	reference group
Men APOE4-	3,274	8,510	0.94	(0.85-1.04)	2.38e-01	3.97e-01
Women APOE4+	6,031	3,344	1.03	(0.93-1.15)	5.34e-01	5.67e-01
Men APOE4+	3,527	2,704	1.07	(0.94-1.21)	3.27e-01	3.68e-01
EADB results (only 65 to 75 yo)						
Women APOE4-	1,903	4,251	1.08	(0.95-1.23)	2.40e-01	reference group
Men APOE4-	1,443	3,391	1.00	(0.86-1.17)	9.61e-01	4.66e-01
Women APOE4+	3,553	1,445	1.01	(0.87-1.17)	9.29e-01	4.77e-01
Men APOE4+	2,143	1,099	0.98	(0.81-1.17)	7.92e-01	3.69e-01
Hutterite results						
All samples	5	26	0.50	(0.44-0.55)	1.89e-14	

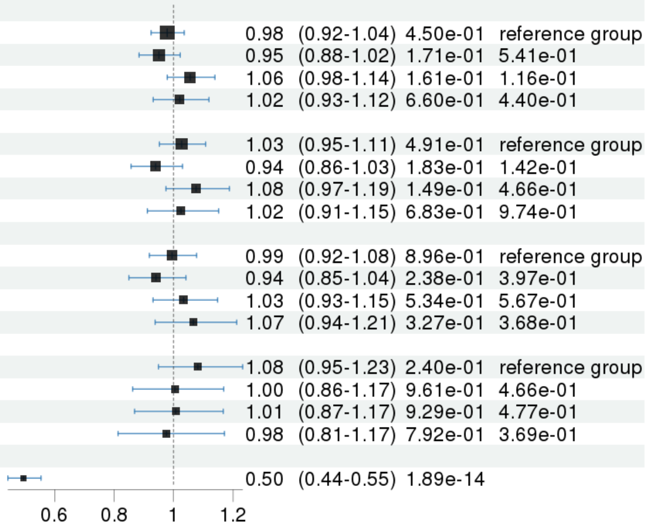

Figure S3: Result of rs2803456 (*MGMT*) in the four sex-*APOEε4* subsets compared with the effect reported in the Hutterite cohort and the effects found in the age-adjusted, *APOEε4* status and younger ages sensitivity analyses. EA: Effect allele, OR: odds ratio, CI: confidence interval, P: p-value, yo: years old.

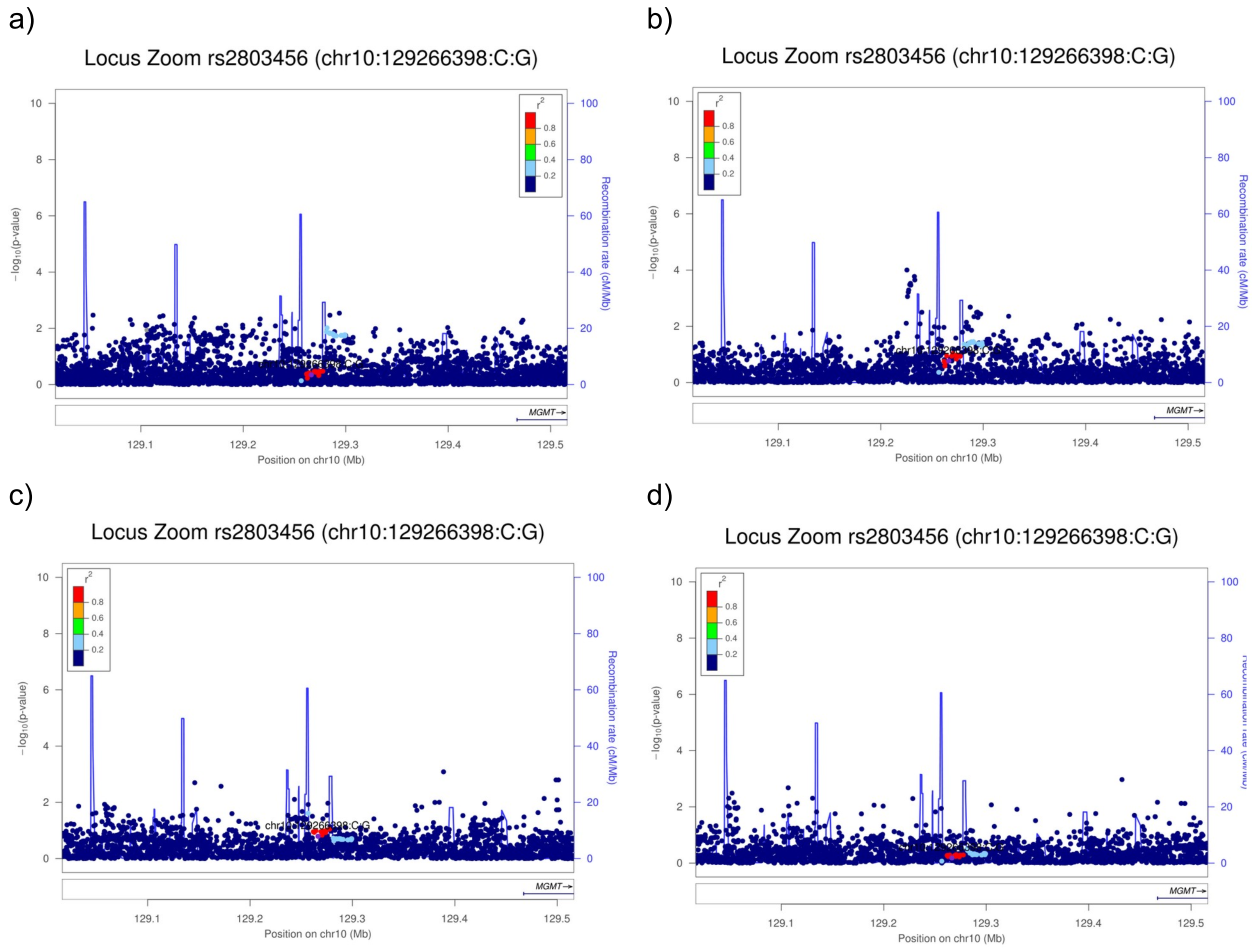

Figure S4: Locus zoom of rs2803456 (*MGMT*) in a) women *APOEε4*-, b) men *APOEε4*-, c) women *APOEε4*+ and d) men *APOEε4*-. Each locus zoom is based on the meta-analysis of the six EADB studies results in one subset. The variant in purple is rs2803456. The positions are in GRCh38 Assembly.

rs12256016 (chr10:129677743:A:G), EA = G

	Cases	Controls	OR	(95% CI)	P	Interaction P
EADB results						
Women APOE4-	10,354	19,910	0.99	(0.95-1.03)	6.27e-01	reference group
Men APOE4-	5,896	16,399	0.97	(0.92-1.02)	2.65e-01	5.69e-01
Women APOE4+	11,047	6,311	0.98	(0.93-1.04)	5.64e-01	8.66e-01
Men APOE4+	6,380	5,538	1.00	(0.94-1.06)	9.62e-01	8.27e-01
EADB results (age-adjusted)						
Women APOE4-	6,704	13,030	0.97	(0.92-1.03)	3.15e-01	reference group
Men APOE4-	4,160	9,464	0.97	(0.91-1.03)	3.61e-01	9.53e-01
Women APOE4+	8,007	3,898	1.01	(0.94-1.08)	8.34e-01	4.37e-01
Men APOE4+	4,856	3,092	1.01	(0.93-1.09)	8.52e-01	4.81e-01
EADB results (no imputed APOE status)						
Women APOE4-	5,519	11,237	0.99	(0.94-1.05)	8.13e-01	reference group
Men APOE4-	3,274	8,510	0.99	(0.93-1.07)	8.54e-01	9.98e-01
Women APOE4+	6,031	3,344	0.96	(0.89-1.03)	2.50e-01	4.39e-01
Men APOE4+	3,527	2,704	1.02	(0.93-1.12)	6.59e-01	6.16e-01
EADB results (only 65 to 75 yo)						
Women APOE4-	1,926	4,380	0.94	(0.85-1.03)	1.65e-01	reference group
Men APOE4-	1,443	3,391	0.95	(0.85-1.06)	3.37e-01	8.46e-01
Women APOE4+	3,553	1,445	1.01	(0.90-1.12)	9.05e-01	3.14e-01
Men APOE4+	2,143	1,099	1.02	(0.90-1.17)	7.13e-01	2.67e-01
Hutterite results						
All samples	5	26	2.02	(1.81-2.26)	1.89e-14	

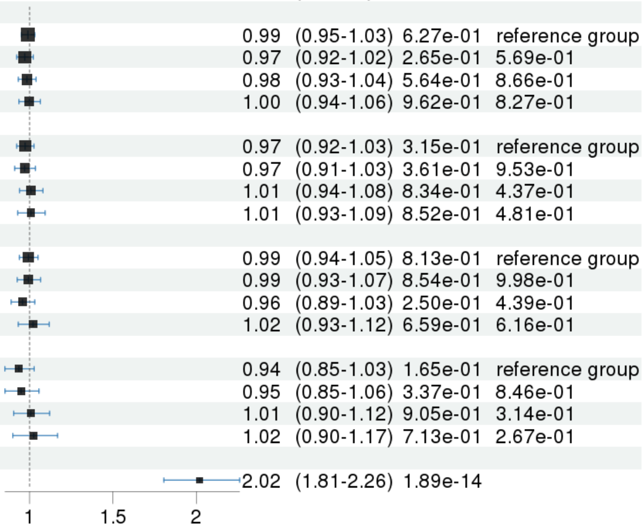

Figure S5: Result of rs12256016 (*MGMT*) in the four sex-*APOEε4* subsets compared with the effect reported in the Hutterite cohort and the effects found in the age-adjusted, *APOEε4* status and younger ages sensitivity analyses. EA: Effect allele, OR: odds ratio, CI: confidence interval, P: p-value, yo: years old.

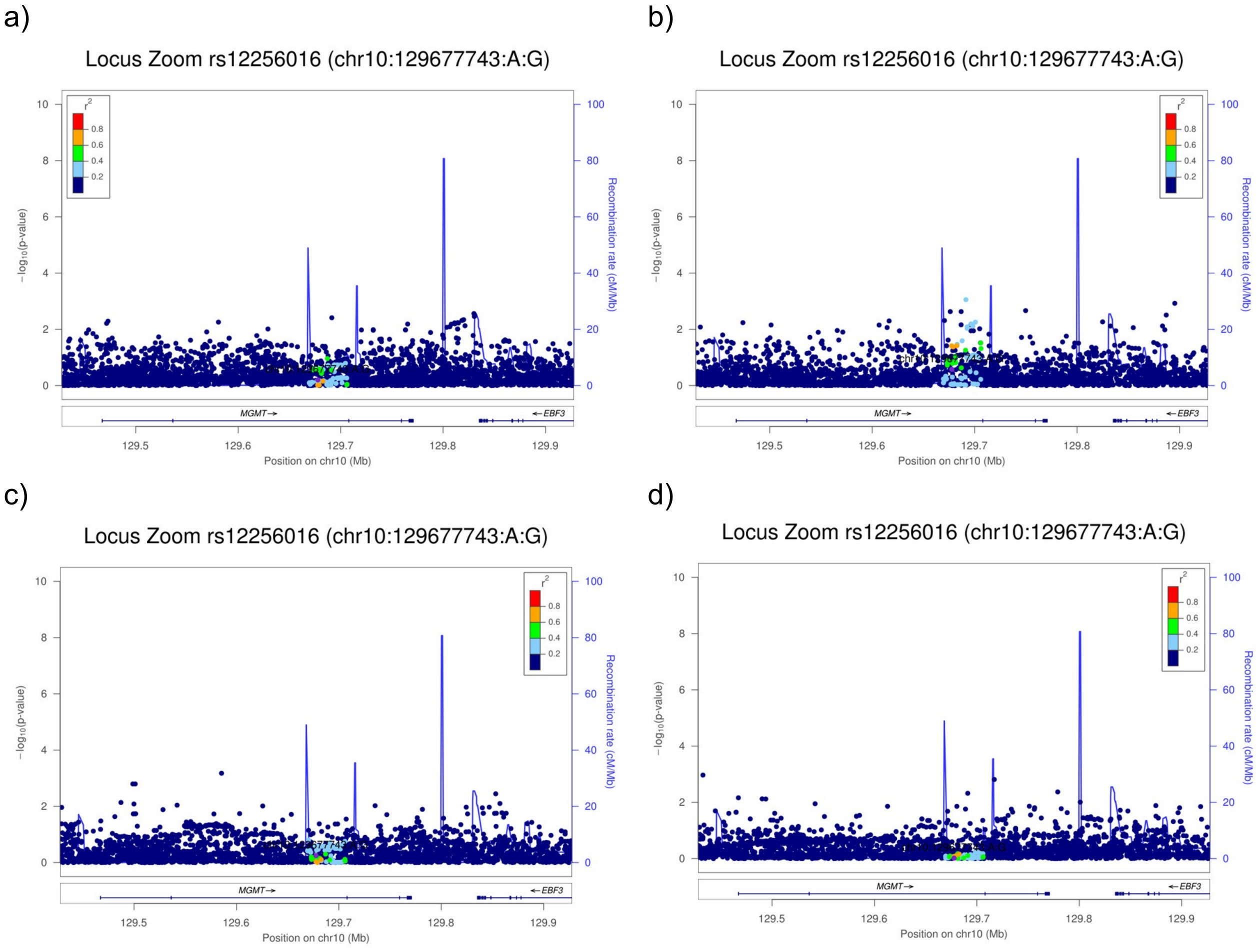

Figure S6: Locus zoom of rs12256016 (*MGMT*) in a) women *APOEε4*-, b) men *APOEε4*-, c) women *APOEε4*+ and d) men *APOEε4*-. Each locus zoom is based on the meta-analysis of the six EADB studies results in one subset. The variant in purple is rs12256016. The positions are in GRCh38 Assembly.

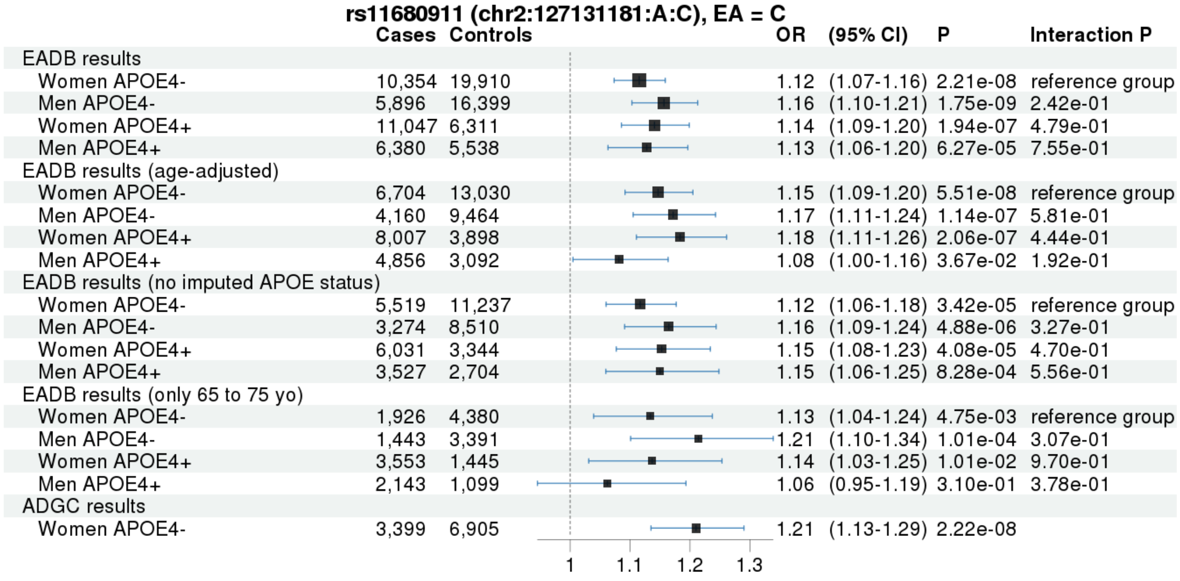

	Cases	Controls	OR	(95% CI)	P	Interaction P
EADB results						
Women APOE4-	10,354	19,910	1.12	(1.07-1.16)	2.21e-08	reference group
Men APOE4-	5,896	16,399	1.16	(1.10-1.21)	1.75e-09	2.42e-01
Women APOE4+	11,047	6,311	1.14	(1.09-1.20)	1.94e-07	4.79e-01
Men APOE4+	6,380	5,538	1.13	(1.06-1.20)	6.27e-05	7.55e-01
EADB results (age-adjusted)						
Women APOE4-	6,704	13,030	1.15	(1.09-1.20)	5.51e-08	reference group
Men APOE4-	4,160	9,464	1.17	(1.11-1.24)	1.14e-07	5.81e-01
Women APOE4+	8,007	3,898	1.18	(1.11-1.26)	2.06e-07	4.44e-01
Men APOE4+	4,856	3,092	1.08	(1.00-1.16)	3.67e-02	1.92e-01
EADB results (no imputed APOE status)						
Women APOE4-	5,519	11,237	1.12	(1.06-1.18)	3.42e-05	reference group
Men APOE4-	3,274	8,510	1.16	(1.09-1.24)	4.88e-06	3.27e-01
Women APOE4+	6,031	3,344	1.15	(1.08-1.23)	4.08e-05	4.70e-01
Men APOE4+	3,527	2,704	1.15	(1.06-1.25)	8.28e-04	5.56e-01
EADB results (only 65 to 75 yo)						
Women APOE4-	1,926	4,380	1.13	(1.04-1.24)	4.75e-03	reference group
Men APOE4-	1,443	3,391	1.21	(1.10-1.34)	1.01e-04	3.07e-01
Women APOE4+	3,553	1,445	1.14	(1.03-1.25)	1.01e-02	9.70e-01
Men APOE4+	2,143	1,099	1.06	(0.95-1.19)	3.10e-01	3.78e-01
ADGC results						
Women APOE4-	3,399	6,905	1.21	(1.13-1.29)	2.22e-08	

Figure S7: Result of rs11680911 (*BIN1*) in the four sex-*APOEε4* subsets compared with the effect reported in ADGC women *APOEε4*- and the effects found in the age-adjusted, *APOEε4* status and younger ages sensitivity analyses. EA: Effect allele, OR: odds ratio, CI: confidence interval, P: p-value, yo: years old.

a)

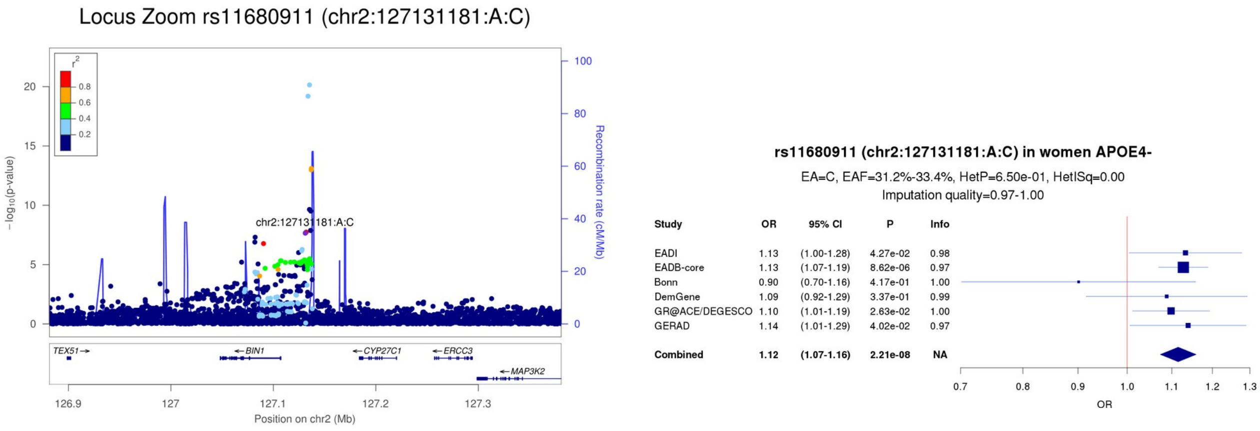
Locus Zoom rs11680911 (chr2:127131181:A:C)
r2
0.8
0.6
0.4
0.2
−log10(p-value)
Recombination rate (cM/Mb)
chr2:127131181:A:C
TEX51
BIN1
CYP27C1
ERCC3
MAP3K2
126.9
127
127.1
127.2
127.3
Position on chr2 (Mb)
rs11680911 (chr2:127131181:A:C) in women APOE4-
EA=C, EAF=31.2%-33.4%, HetP=6.50e-01, HetISq=0.00
Imputation quality=0.97-1.00
Study OR 95% CI P Info
EADI 1.13 (1.00-1.28) 4.27e-02 0.98
EADB-core 1.13 (1.07-1.19) 8.62e-06 0.97
Bonn 0.90 (0.70-1.16) 4.17e-01 1.00
DemGene 1.09 (0.92-1.29) 3.37e-01 0.99
GR@ACE/DEGESCO 1.10 (1.01-1.19) 2.63e-02 1.00
GERAD 1.14 (1.01-1.29) 4.02e-02 0.97
Combined 1.12 (1.07-1.16) 2.21e-08 NA
0.7 0.8 0.9 1.0 1.1 1.2 1.3
OR

b)

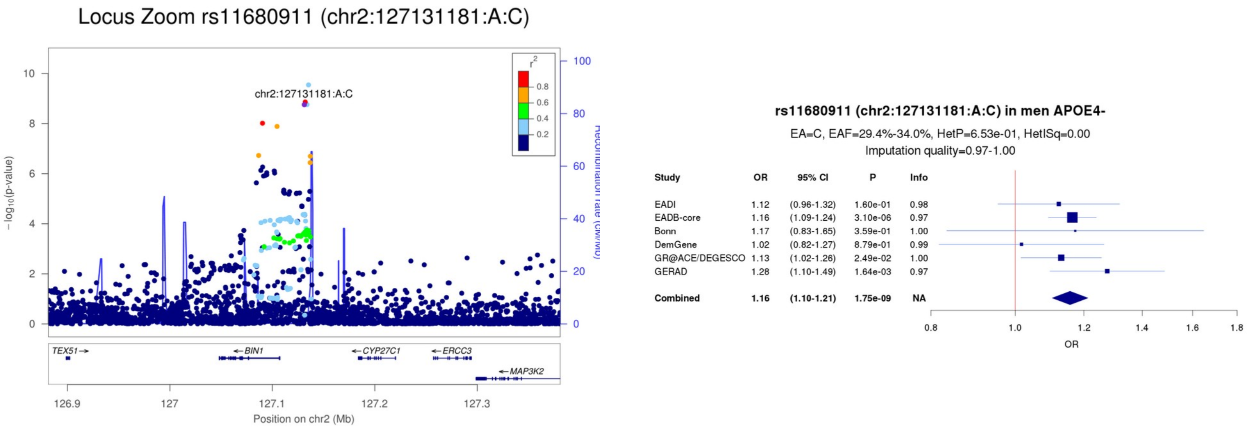
Locus Zoom rs11680911 (chr2:127131181:A:C)
r2
0.8
0.6
0.4
0.2
−log10(p-value)
Recombination rate (cM/Mb)
chr2:127131181:A:C
TEX51
BIN1
CYP27C1
ERCC3
MAP3K2
126.9
127
127.1
127.2
127.3
Position on chr2 (Mb)
rs11680911 (chr2:127131181:A:C) in men APOE4-
EA=C, EAF=29.4%-34.0%, HetP=6.53e-01, HetISq=0.00
Imputation quality=0.97-1.00
Study OR 95% CI P Info
EADI 1.12 (0.96-1.32) 1.60e-01 0.98
EADB-core 1.16 (1.09-1.24) 3.10e-06 0.97
Bonn 1.17 (0.83-1.65) 3.59e-01 1.00
DemGene 1.02 (0.82-1.27) 8.79e-01 0.99
GR@ACE/DEGESCO 1.13 (1.02-1.26) 2.49e-02 1.00
GERAD 1.28 (1.10-1.49) 1.64e-03 0.97
Combined 1.16 (1.10-1.21) 1.75e-09 NA
0.8 1.0 1.2 1.4 1.6 1.8
OR

c)

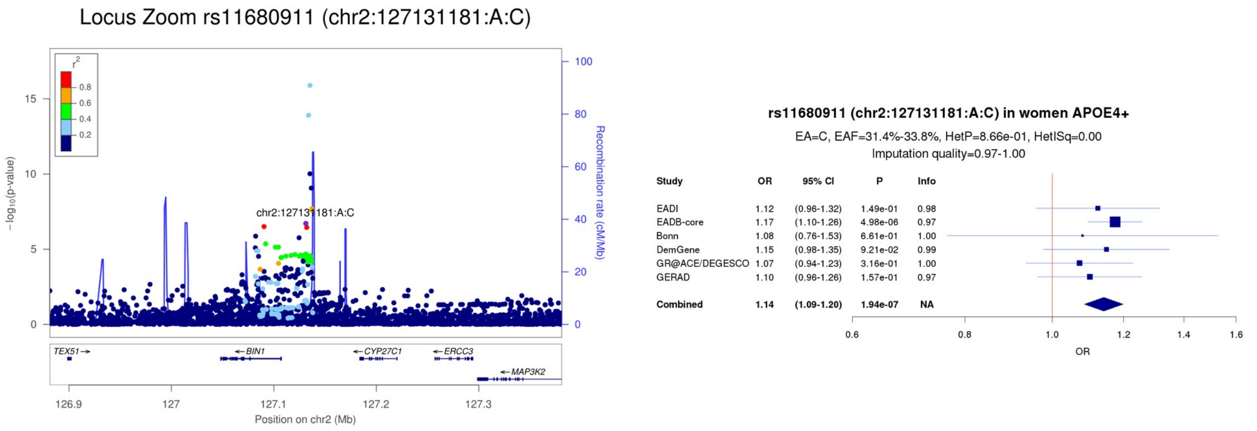
Locus Zoom rs11680911 (chr2:127131181:A:C)
r2
0.8
0.6
0.4
0.2
−log10(p-value)
Recombination rate (cM/Mb)
chr2:127131181:A:C
TEX51
BIN1
CYP27C1
ERCC3
MAP3K2
126.9
127
127.1
127.2
127.3
Position on chr2 (Mb)
rs11680911 (chr2:127131181:A:C) in women APOE4+
EA=C, EAF=31.4%-33.8%, HetP=8.66e-01, HetISq=0.00
Imputation quality=0.97-1.00
Study OR 95% CI P Info
EADI 1.12 (0.96-1.32) 1.49e-01 0.98
EADB-core 1.17 (1.10-1.26) 4.98e-06 0.97
Bonn 1.08 (0.76-1.53) 6.61e-01 1.00
DemGene 1.15 (0.98-1.35) 9.21e-02 0.99
GR@ACE/DEGESCO 1.07 (0.94-1.23) 3.16e-01 1.00
GERAD 1.10 (0.96-1.26) 1.57e-01 0.97
Combined 1.14 (1.09-1.20) 1.94e-07 NA
0.6 0.8 1.0 1.2 1.4 1.6
OR

d)

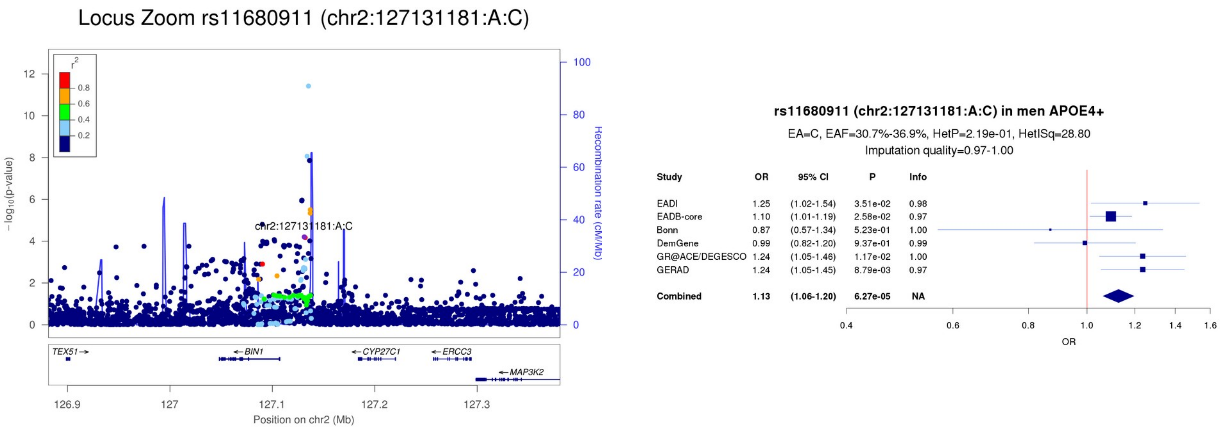

Figure S8: Locus zoom and forest plots of rs11680911 (*BIN1*) in a) women *APOEε4*-, b) men *APOEε4*-, c) women *APOEε4+* and d) men *APOEε4*-. Each locus zoom is based on the meta-analysis of the six EADB studies results in one subset. The variant in purple is rs11680911. The positions are in GRCh38 Assembly. OR: odds ratio, CI: confidence interval, EA: effect allele, EAF: effect allele frequency range across all studies, HetP: heterogeneity P value, HetISq: heterogeneity statistic, info: imputation quality.

rs138350418 (chr10:129830097:G:A), EA = A

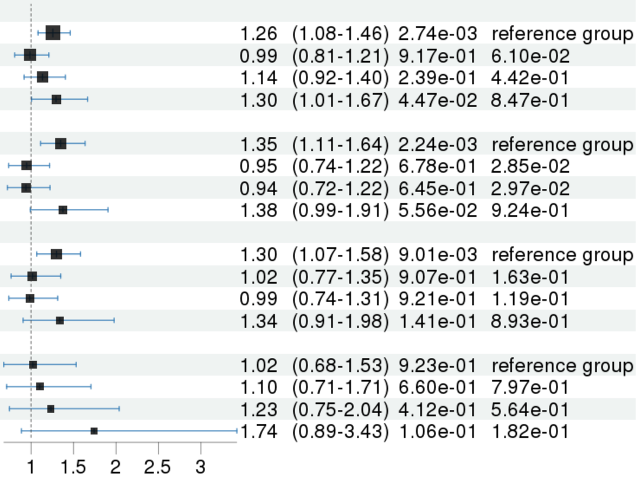

	Cases	Controls	OR	(95% CI)	P	Interaction P
EADB results						
Women APOE4-	10,354	19,910	1.26	(1.08-1.46)	2.74e-03	reference group
Men APOE4-	5,788	15,988	0.99	(0.81-1.21)	9.17e-01	6.10e-02
Women APOE4+	10,847	6,157	1.14	(0.92-1.40)	2.39e-01	4.42e-01
Men APOE4+	6,273	5,406	1.30	(1.01-1.67)	4.47e-02	8.47e-01
EADB results (age-adjusted)						
Women APOE4-	6,704	13,030	1.35	(1.11-1.64)	2.24e-03	reference group
Men APOE4-	4,052	9,062	0.95	(0.74-1.22)	6.78e-01	2.85e-02
Women APOE4+	7,809	3,747	0.94	(0.72-1.22)	6.45e-01	2.97e-02
Men APOE4+	4,749	2,961	1.38	(0.99-1.91)	5.56e-02	9.24e-01
EADB results (no imputed APOE status)						
Women APOE4-	5,519	11,237	1.30	(1.07-1.58)	9.01e-03	reference group
Men APOE4-	3,166	8,102	1.02	(0.77-1.35)	9.07e-01	1.63e-01
Women APOE4+	5,834	3,192	0.99	(0.74-1.31)	9.21e-01	1.19e-01
Men APOE4+	3,421	2,574	1.34	(0.91-1.98)	1.41e-01	8.93e-01
EADB results (only 65 to 75 yo)						
Women APOE4-	1,568	3,386	1.02	(0.68-1.53)	9.23e-01	reference group
Men APOE4-	1,158	2,584	1.10	(0.71-1.71)	6.60e-01	7.97e-01
Women APOE4+	2,373	999	1.23	(0.75-2.04)	4.12e-01	5.64e-01
Men APOE4+	1,458	828	1.74	(0.89-3.43)	1.06e-01	1.82e-01

Figure S9: Result of rs138350418 (*MGMT*) in the four sex-*APOEε4* subsets compared with the effects found in the age-adjusted, *APOEε4* status and younger ages sensitivity analyses. EA: Effect allele, OR: odds ratio, CI: confidence interval, P: p-value, yo: years old.

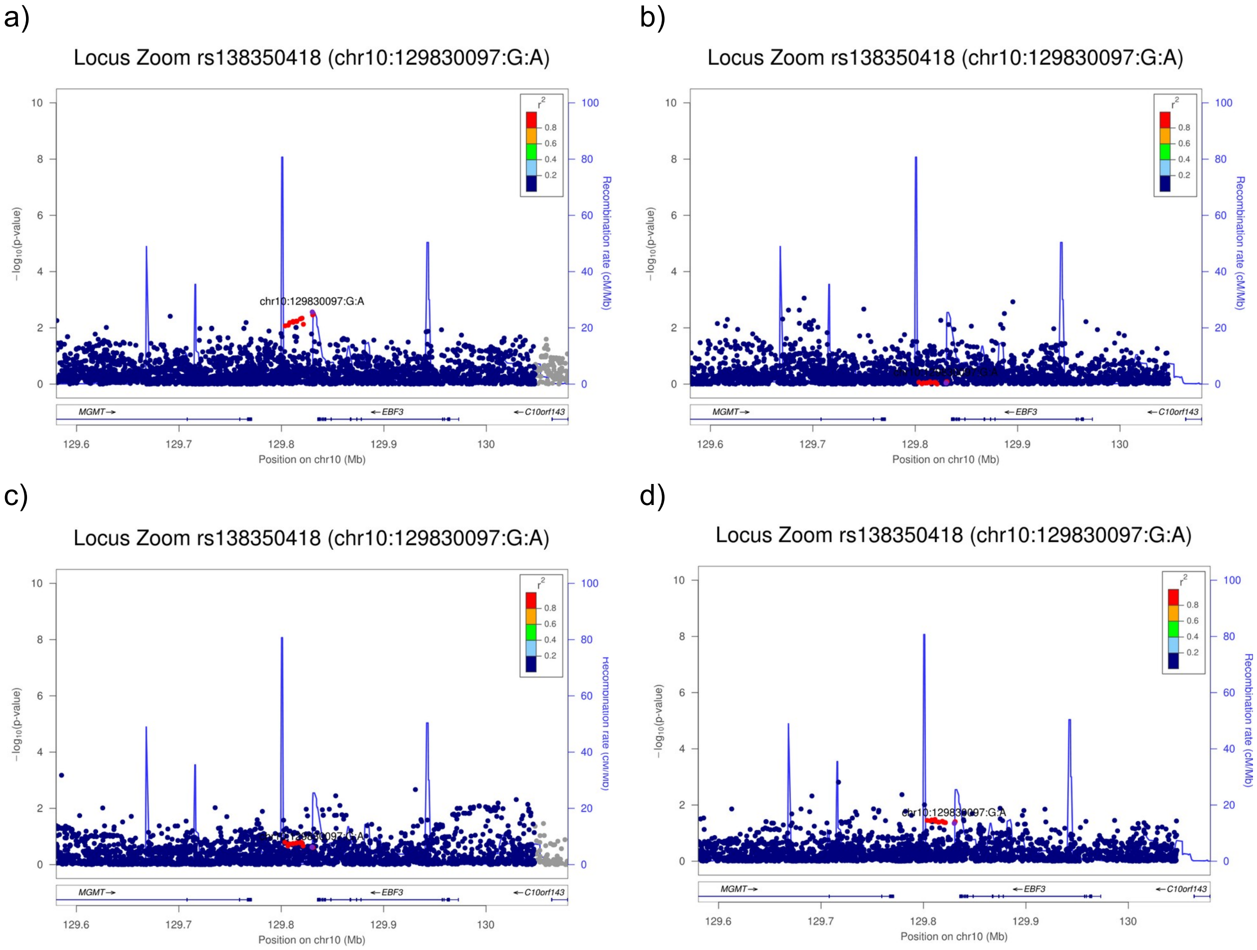

Figure S10: Locus zoom of rs138350418 (*MGMT*) in a) women *APOEε4*-, b) men *APOEε4*-, c) women *APOEε4*+ and d) men *APOEε4*-. Each locus zoom is based on the meta-analysis of the six EADB studies results in one subset. The variant in purple is rs138350418. The positions are in GRCh38 Assembly.

rs6733839 (chr2:127135234:C:T), EA = T

	Cases	Controls	OR	(95% CI)	P	Interaction P
EADB results						
Women APOE4-	10,354	19,910	1.19	(1.15-1.24)	7.15e-21	reference group
Men APOE4-	5,896	16,399	1.16	(1.11-1.21)	2.84e-10	3.37e-01
Women APOE4+	11,047	6,311	1.22	(1.17-1.28)	1.28e-16	3.81e-01
Men APOE4+	6,380	5,538	1.22	(1.16-1.29)	3.79e-12	4.66e-01
EADB results (age-adjusted)						
Women APOE4-	6,704	13,030	1.20	(1.15-1.26)	2.29e-14	reference group
Men APOE4-	4,160	9,464	1.17	(1.11-1.24)	2.36e-08	5.22e-01
Women APOE4+	8,007	3,898	1.26	(1.19-1.34)	1.01e-13	2.30e-01
Men APOE4+	4,856	3,092	1.23	(1.15-1.32)	6.28e-09	5.77e-01
EADB results (no imputed APOE status)						
Women APOE4-	5,519	11,237	1.20	(1.14-1.26)	1.65e-12	reference group
Men APOE4-	3,274	8,510	1.17	(1.10-1.24)	1.36e-06	5.35e-01
Women APOE4+	6,031	3,344	1.22	(1.14-1.31)	2.67e-09	6.47e-01
Men APOE4+	3,527	2,704	1.24	(1.14-1.34)	1.58e-07	5.00e-01
EADB results (only 65 to 75 yo)						
Women APOE4-	1,926	4,380	1.23	(1.14-1.34)	8.81e-07	reference group
Men APOE4-	1,443	3,391	1.20	(1.09-1.32)	2.30e-04	6.33e-01
Women APOE4+	3,553	1,445	1.23	(1.12-1.35)	9.40e-06	9.91e-01
Men APOE4+	2,143	1,099	1.16	(1.04-1.30)	8.72e-03	3.81e-01

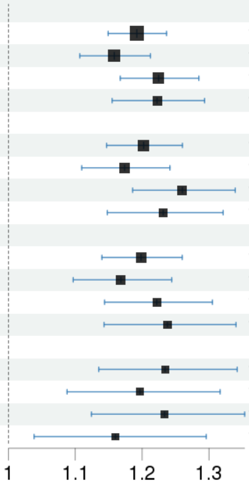

Figure S11: Result of rs6733839 (*BIN1*) in the four sex-*APOEε4* subsets compared with the effects found in the age-adjusted, *APOEε4* status and younger ages sensitivity analyses. EA: Effect allele, OR: odds ratio, CI: confidence interval, P: p-value, yo: years old.

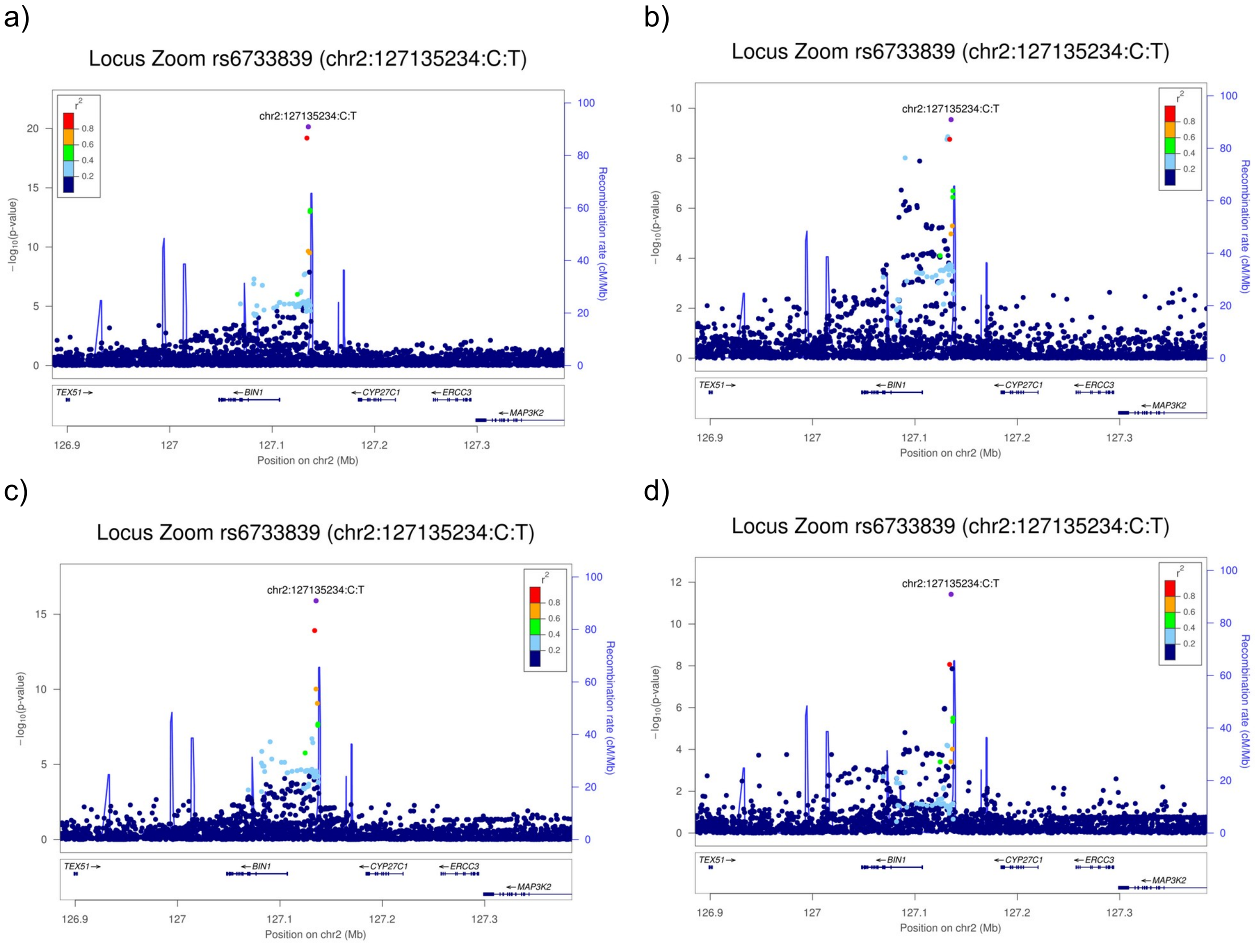

Figure S12: Locus zoom of rs6733839 (*BIN1*) in a) women *APOEε4*-, b) men *APOEε4*-, c) women *APOEε4*+ and d) men *APOEε4*-. Each locus zoom is based on the meta-analysis of the six EADB studies results in one subset. The variant in purple is rs6733839. The positions are in GRCh38 Assembly.

8) Consortia author lists

EADB

Céline Bellenguez[1], Fahri Küçükali[2,3,4], Iris Jansen[5,6], Victor Andrade[7,8], Sonia Moreno-Grau[9,10], Najaf Amin[11,12], Benjamin Grenier-Boley[1], Rafael Campos-Martin[7], Peter A. Holmans[13], Anne Boland[14], Luca Kleineidam[7,8,15], Vincent Damotte[1], Sven J. van der Lee[5,16], Teemu Kuulasmaa[17], Itziar de Rojas[9,10], Amber Yaqub[11], Ivana Prokic[11], Marcos R, Costa[1,18], Julien Chapuis[1], Shahzad Ahmad[11,19], Vilmantas Giedraitis[20], Dag Aarsland[21,22], Pablo Garcia-Gonzalez[9,10], Carla Abdelnour[9,10], Emilio Alarcón-Martín[9,23], Daniel Alcolea[10,24], Montserrat Alegret[9,10], Ignacio Alvarez[25,26], Victoria Álvarez[27,28], Nicola J. Armstrong[29], Tsolaki Anthoula[30,31], Ildebrando Appollonio[32,33], Marina Arcaro[34], Silvana Archetti[35], Alfonso Arias Pastor[36,37], Beatrice Arosio[38,39], Lavinia Athanasiu[40], Henri Bailly[41], Nerisa Banaj[42], Miquel Baquero[43], Ana Belén Pastor[44], Luisa Benussi[45], Claudine Berr[46], Céline Besse[14], Valentina Bessi[47,48], Giuliano Binetti[45,49], Alessandra Bizarro[50], Rafael Blesa[10,24], Mercè Boada[9,10], Barbara Borroni[51], Silvia Boschi[52], Paola Bossù[53], Geir Bråthen[54,55], Catherine Bresner[13], Henry Brodaty[29,56], Keeley J. Brookes[57], Luis Ignacio Brusco[58,59,60], Dolores Buiza-Rueda[10,61], Katharina Bûrger[62,63], Vanessa Burholt[64,65], Miguel Calero[10,44,66], Geneviève Chene[67,68], Ángel Carracedo[69,70], Roberta Cecchetti[71], Laura Cervera-Carles[10,24], Camille Charbonnier[72], Caterina Chillotti[73], Simona Ciccone[39], Jurgen A.H.R. Claassen[74], Jordi Clarimon[10,24], Christopher Clark[75], Elisa Conti[32], Anaïs Corma-Gómez[76], Emanuele Costantini[77], Carlo Custodero[78], Delphine Daian[14], Maria Carolina Dalmasso[7], Antonio Daniele[77], Efthimios Dardiotis[79], Jean-François Dartigues[80], Peter Paul de Deyn[81], Stéphanie Debette[80,82], Jürgen Deckert[83], Teodoro del Ser[44], Nicola Denning[84], Martin Dichgans[62,63,85], Janine Diehl-Schmid[86], Mónica Diez-Fairen[25,26], Paolo Dionigi Rossi[39], Srdjan Djurovic[40], Emmanuelle Duron[41], Emrah Düzel[87,88], Carole Dufouil[67,68], Valentina Escott-Price[13,84], Ana Espinosa[9,10], Michael Ewers[62,63], Marta Fernández-Fuertes[76], Catarina B Ferreira[89], Evelyn Ferri[39], Bertrand Fin[14], Peter Fischer[90], Tormod Fladby[91], Klaus Fließbach[8,15], Juan Fortea[10,24], Silvia Fostinelli[45], Nick C. Fox[92], Emlio Franco-Macías[93], María J. Bullido[10,94,95], Ana Frank-García[10,94,96], Lutz Froelich[97], Daniela Galimberti[34,88], Jose Maria García-Alberca[10,98], Pablo García-González[9], Sebastian Garcia-Madrona[99], Guillermo Garcia-Ribas[99], Roberta Ghidoni[45], Ina Giegling[100], Giaccone Giorgio[85], Oliver Goldhardt[86], Antonio González-Péréz[101], Caroline Graff[102,118], Giulia Grande[103], Emma Green[104], Timo Grimmer[86], Edna Grünblatt[105,106,107], Tamar Guetta-Baranes[108], Annakaisa Haapasalo[109], Georgios Hadjigeorgiou[110], Harald Hampel[111,112], Olivier Hanon[41], John Hardy[113], Annette M. Hartmann[100], Lucrezia Hausner[97], Janet Harwood[13], Stefanie Heilmann-Heimbach[114], Seppo Helisalmi[115,116], Michael T. Heneka[8,16], Isabel Hernández[9,10], Martin J. Herrmann[83], Per Hoffmann[114], Clive Holmes[117], Henne Holstege[5,16], Raquel Huerto Vilas[36,37], Marc Hulsman[5,16], Charlotte Johansson[102,118], Lena Kilander[20], Anne Kinhult Ståhlbom[102,118], Miia Kivipelto[119,120,121,122], Anne Koivisto[115], Johannes Kornhuber[123], Mary H. Kosmidis[124], Carmen Lage[10,125], Erika J Laukka[103,126], Alessandra Lauria[50], Jenni Lehtisalo[115,127], Ondrej Lerch[128,129], Alberto Lleó[10,24], Adolfo Lopez de Munain[10,130], Malin Löwemark[20], Lauren Luckcuck[13], Juan Macías[76], Catherine A. MacLeod[131], Wolfgang Maier[8,15], Francesca Mangialasche[119], Spallazzi Marco[132], Marta Marquié[9,10], Rachel Marshall[13], Angel Martín Montes[10,94,96], Carmen Martínez Rodríguez[28], Carlo Masullo[133], Simon Mead[134], Patrizia Mecocci[71], Miguel Medina[10,44], Alun Meggy[84], Shima Mehrabian[135], Silvia Mendoza[98], Manuel Menéndez-González[28], Pablo Mir[10,61], Susanne Moebus[136], Merel Mol[137], Laura Molina-Porcel[138,139], Laura Montrreal[9], Laura Morelli[140], Fermin Moreno[10,130], Kevin Morgan[141], Markus M Möthen[114], Carolina Muchnik[58], Benedetta Nacmias[47,142], Tiia Ngandu[127], Gael Nicolas[72], Børge G. Nordestgaard[143,144], Robert Olaso[14], Adelina Orellana[9,10], Michela Orsini[77], Gemma Ortega[9,10], Alessandro Padovani[51], Caffarra Paolo[145], Goran Papenberg[103], Lucilla Parnetti[87], Pau Pastor[25,26], Alba Pérez-Cordón[9], Jordi Pérez-Tur[10,146,147], Pierre Pericard[148], Oliver Peters[149,150], Yolande A.L. Pijnenburg[5], Juan A Pineda[76], Gerard Piñol-Ripoll[36,37], Claudia Pisanu[151], Thomas Polak[83], Julius

Popp[152,153,154], Danielle Posthuma[6], Josef Priller[150,155], Raquel Puerta[9], Olivier Quenez[72], Inés Quintela[69], Jesper Qvist Thomassen[156], Alberto Rábano[10,44], Innocenzo Rainero[52], Inez Ramakers[157], Luis M Real[76,158], Marcel J.T. Reinders[159], Steffi Riedel-Heller[160], Peter Riederer[161], Natalia Roberto[9], Eloy Rodriguez-Rodriguez[10,125], Arvid Rongve[162,163], Irene Rosas Allende[27,28], Maitée Rosende-Roca[9,10], Jose Luis Royo[164], Elisa Rubino[165], Dan Rujescu[100], María Eugenia Sáez[101], Paraskevi Sakka[166], Ingvild Saltvedt[55,167], Ángela Sanabria[9,10], María Bernal Sánchez-Arjona[93], Florentino Sanchez-Garcia[168], Pascual Sánchez Juan[10,125], Raquel Sánchez-Valle[169], Sigrid B Sando[54,55], Michela Scamosci[71], Nikolaos Scarmeas[170,171], Elio Scarpini[34,88], Philip Scheltens[5], Norbert Scherbaum[172], Martin Scherer[173], Matthias Schmid[16,174], Anja Schneider[8,16], Jonathan M. Schott[92], Geir Selbæk[91,175], Davide Seripa[176], Alexey A Shadrin[40], Olivia Skrobot[119], Hilkka Soininen[115], Vincenzo Solfrizzi[78], Alina Solomon[115], Sandro Sorbi[47,142], Oscar Sotolongo-Grau[9], Gianfranco Spalletta[42], Annika Spottke[16], Alessio Squassina[177], Eystein Stordal[178], Juan Pablo Tartan[9], Lluís Tárraga[9,10], Niccolo Tesí[5,16], Anbupalam Thalamuthu[29], Tegos Thomas[30,31], Latchezar Traykov[135], Lucio Tremolizzo[32,33], Anne Tybjærg-Hansen[144,156], Andre Uitterlinden[179], Abbe Ullgren[102], Ingun Ulstein[175], Sergi Valero[9,10], Aad van der Lugt[180], Jasper Van Dongen[2,3,4], Jeroen van Rooij[137], John van Swieten[137], Rik Vandenberghe[181,182], Frans Verhey[157], Jean-Sébastien Vidal[41], Jonathan Vogelgsang[183,184], Martin Vyhnalek[128,129], Michael Wagner[8,16], David Wallon[185], Leonie Weinhold[174], Jens Wiltfang[183,186,187], Gill Windle[131], Bob Woods[131], Mary Yannakoulia[188], Miren Zulaica[10,189], Jan Laczo[128,129], Vaclav Matoska[190], Maria Serpente[88], Francesca Assogna[42], Fabrizio Piras[42], Federica Piras[42], Valentina Ciullo[42], Jacob Shofany[42], Carlo Ferrarese[32,33], Simona Andreoni[32], Gessica Sala[32], Chiara Paola Zoia[32], Maria Del Zompo[177], Alberto Benussi[51], Patrizia Bastiani[191], Mari Takalo*[192], Teemu Natunen*[192], Tiina Laatikainen[120,127], Jaakko Tuomilehto[120,127], Riitta Antikainen[193,194], Timo Strandberg[193,195], Jaana Lindström[127], Markku Peltonen[127], Richard Abraham[196], Ammar Al-Chalabi[197], Nicholas J. Bass[198], Carol Brayne[199], Kristelle S. Brown[200], John Collinge[201], David Craig[202], Pangiotis Deloukas[203], Nick Fox[204], Amy Gerrish[204], Michael Gill[205], Rhian Gwilliam[203], John Hardy[206], Denise Harold[207], Paul Hollingworth[196], Jarret A, Johnston[208], Lesley Jones[196], Brian Lawlor[205], Gill Livingston[198], Simon Lovestone[209], Michelle Lupton[210,211], Aoibhinn Lynch[205], David Mann[212], Bernadette McGuinness[208], Andrew McQuillin[198], Michael C. O'Donovan[196], Michael J. Owen[196], Peter Passmore[208], John F, Powell[210,211], Petra Proitsi[210,211], Martin Rossor[204], Christopher E. Shaw[197], A. David Smith[213], Hugh Gurling[214], Stephen Todd[215], Catherine Mummery[216], Nathalie Ryan[216], Giordano Lacidogna[77], Ad Adarmes-Gómez[10,61], Ana Mauleón[9], Ana Pancho[9], Anna Gailhajenet[9], Asunción Lafuente[9], D Macias-García[10,61], Elvira Martín[9], Esther Pelejà[9], F Carrillo[10,61], Isabel Sastre Merlín[10,95], L Garrote-Espina[10,61], Liliana Vargas[9], M Carrion-Claro[10,61], M Marín[93], Ma Labrador[10,61], Mar Buendia[9], María Dolores Alonso[217], Marina Guitart[9], Mariona Moreno[9], Marta Ibarria[9], Mt Periñán[10,61], Nuria Aguilera[9], P Gómez-Garre[10,61], Pilar Cañabate[9], R Escuela[10,61], R Pineda-Sánchez[10,61], R Vigo-Ortega[10,61], S Jesús[10,61], Silvia Preckler[9], Silvia Rodrigo-Herrero[93], Susana Diego[9], Alessandro Vacca[52], Fausto Roveta[52], Nicola Salvadori[87], Elena Chipi[87], Henning Boecker[15,218], Christoph Laske[219,220], Robert Perneczky[65,221], Costas Anastasiou[188], Daniel Janowitz[62], Rainer Malik[62], Anna Anastasiou[30], Kayenat Parveen[7], Carmen Lage[222], Sara López-García[222], Anna Antonell[169], Kalina Yonkova Mihova[223], Diyana Belezhanska[135], Heike Weber[224], Silvia Kochen[225], Patricia Solis[225], Nancy Medel[225], Julieta Lisso[225], Zulma Sevillano[225], Daniel G Politis[225,226], Valeria Cores[225,226], Carolina Cuesta[225,226], Cecilia Ortiz[227], Juan Ignacio Bacha[227], Mario Rios[228], Aldo Saenz[228], Mariana Sanchez Abalos[229], Eduardo Kohler[230], Dana Lis Palacio[231], Ignacio Etchepareborda[231], Matias Kohler[231], Gisela Novack[232], Federico Ariel Prestia[232], Pablo Galeano[232], Eduardo M. Castaño[232], Sandra Germani[233], Carlos Reyes Toso[233], Matias Rojo[233], Carlos Ingino[233], Carlos Mangone[233], Sebastiaan Engelborghs[234,235,236,237], Tagliavini Fabrizio[238], Sune Fallgaard Nielsen[239], Lucia Farotti[240], Chiara Fenoglio[241], Geert Jan Biessels[242], Seth Love[243], Patrick G. Kehoe[243], Florence Pasquier[244], Christine Van Broeckhoven[2,3,245], David C. Rubinsztein[246], Stefan Teipel[247], Nathalie

Fievet[1], Vincent Deramecourt[244], Charlotte Forsell[102,118], Håkan Thonberg[102,118], Maria Bjerke[69], Ellen De Roeck[69], María Teresa Martínez-Larrad[2248], Natividad Olivar[233], Mohsen Ghanbari[11], Perminder Sachdev[29], Karen Mather[29], Frank Jessen[8,16], M. Arfan Ikram[11], Alexandre de Mendonça[89], Jakub Hort[128,129], Tsolaki Magda[30,31], Philippe Amouyel[1], Julie Williams[13], Ruth Frikke-Schmidt[144,156], Jordi Clarimon[10,24], Jean-François Deleuze[14], Giacomina Rossi[85], Ole A. Andreassen[40], Martin Ingelsson[20], Mikko Hiltunen[17], Kristel Sleegers[2,3,4], Cornelia M. van Duijn[11,12], Rebecca Sims[13], Wiesje M. van der Flier[5], Agustín Ruiz[9,10], Alfredo Ramirez[7,8,16,249], Jean-Charles Lambert[1]

1. Univ. Lille, Inserm, CHU Lille, Institut Pasteur de Lille, U1167-RID-AGE facteurs de risque et déterminants moléculaires des maladies liés au vieillissement, Lille, France
2. Complex Genetics of Alzheimer's Disease Group, VIB Center for Molecular Neurology, VIB, Antwerp, Belgium
3. Laboratory of Neurogenetics, Institute Born - Bunge, Antwerp, Belgium
4. Department of Biomedical Sciences, University of Antwerp, Neurodegenerative Brain Diseases Group,
5. Alzheimer Center Amsterdam, Department of Neurology, Amsterdam Neuroscience, Vrije Universiteit Amsterdam, Amsterdam UMC, Amsterdam, The Netherlands
6. Department of Complex Trait Genetics, Center for Neurogenomics and Cognitive Research, Amsterdam, The Netherlands
7. Division of Neurogenetics and Molecular Psychiatry, Department of Psychiatry and Psychotherapy, University of Cologne, Medical Faculty, Cologne, Germany.
8. Department of Neurodegenerative Diseases and Geriatric Psychiatry, University Hospital Bonn, Bonn, Germany
9. Research Center and Memory clinic Fundació ACE, Institut Català de Neurociències Aplicades, Universitat Internacional de Catalunya, Barcelona, Spain
10. CIBERNED, Network Center for Biomedical Research in Neurodegenerative Diseases, National Institute of Health Carlos III, Madrid, Spain
11. Department of Epidemiology, ErasmusMC, Totterdam, The Netherlands
12. Nuffield Department of Population Health Oxford University, Oxford, UK
13. MRC Centre for Neuropsychiatric Genetics and Genomics, , Division of Psychological Medicine and Clinical
14. Université Paris-Saclay, CEA, Centre National de Recherche en Génomique Humaine, 91057, Evry, France
15. German Center for Neurodegenerative Diseases (DZNE Bonn), Bonn, Germany
16. Section Genomics of Neurdegenerative Diseases and Aging, Department of Human Genetics Amsterdam, The Netherlands
17. Institute of Biomedicine, University of Eastern Finland, Kuopio, Finland
18. Brain Institute, Federal University of Rio Grande do Norte, Av. Nascimento de Castro 2155 Natal, Brazil
19. LACDR, Leiden, The Netherlands
20. Dept.of Public Health and Carins Sciences / Geriatrics, Uppsala University, Sweden
21. Centre of Age-Related Medicine, Stavanger University Hospital, Norway
22. Institute of Psychiatry, Psychology & Neuroscience, PO 70, 16 De Crespigny Park, London, UK
23. Department of Surgery, Biochemistry and Molecular Biology, School of Medicine, University of Málaga, Málaga, Spain.
24. Department of Neurology, II B Sant Pau, Hospital de la Santa Creu i Sant Pau, Universitat Autònoma de Barcelona, Barcelona, Spain.
25. Fundació Docència i Recerca MútuaTerrassa and Movement Disorders Unit, Department of Neurology, University Hospital MútuaTerrassa, Terrassa 08221, Barcelona, Spain
26. Memory Disorders Unit, Department of Neurology, Hospital Universitari Mutua de Terrassa, Terrassa, Barcelona, Spain.
27. Laboratorio de Genética. Hospital Universitario Central de Asturias, Oviedo, Spain
28. Servicio de Neurología HOspital Universitario Central de Asturias- Oviedo and Instituto de Investigación Biosanitaria del Principado de Asturias, Oviedo, Spain

29. Centre for Healthy Brain Ageing, School of Psychiatry, Faculty of Medicine, University of New South Wales, Sydney, Australia
30. 1st Department of Neurology, Medical school, Aristotle University of Thessaloniki, Thessaloniki, Makedonia, Greece
31. Alzheimer Hellas, Thessaloniki, Makedonia, Greece
32. School of Medicine and Surgery, University of Milano-Bicocca, Italy
33. Neurology Unit, "San Gerardo" hospital, Monza, Italy
34. Fondazione IRCCS Ca' Granda, Ospedale Policlinico, Milan, Italy
35. Department of Laboratory Diagnostics, III Laboratory of Analysis, Brescia Hospital, Brescia, Italy
36. Unitat Trastorns Cognitius, Hospital Universitari Santa Maria de Lleida, Lleida, Spain
37. Institut de Recerca Biomedica de Lleida (IRBLLeida), Lleida, Spain
38. Department of Clinical Sciences and Community Health, University of Milan, Italy
39. Geriatic Unit, Fondazione Cà Granda, IRCCS Ospedale Maggiore Policlinico, Milan, Italy
40. NORMENT Centre, University of Oslo, Oslo, Norway
41. Université de Paris, EA 4468, APHP, Hôpital Broca, Paris, France
42. Laboratory of Neuropsychiatry, Department of Clinical and Behavioral Neurology, IRCCS Santa Lucia Foundation, Rome, Italy
43. Servei de Neurologia, Hospital Universitari i Politècnic La Fe, Valencia, Spain.
44. CIEN Foundation/Queen Sofia Foundation Alzheimer Center, Madrid, Spain
45. Molecular Markers Laboratory, IRCCS Istituto Centro San Giovanni di Dio Fatebenefratelli, Brescia, Italy
46. Univ. Montpellier, Inserm U1061, Neuropsychiatry: epidemiological and clinical research, PSNREC, Montpellier, France
47. Department of Neuroscience, Psychology, Drug Research and Child Health University of Florence, Florence Italy
48. Azienda Ospedaliero-Universitaria Careggi, Florence, Italy
49. MAC - Memory Clinic, IRCCS Istituto Centro San Giovanni di Dio Fatebenefratelli, Brescia
50. Geriatrics Unit Fondazione Policlinico A. Gemelli IRCCS, Rome , Italy
51. Centre for Neurodegenerative Disorders, Department of Clinical and Experimental Sciences, University of Brescia, Brescia, Italy
52. Department of Neuroscience “Rita Levi Montalcini”, University of Torino, Torino, Italy
53. Experimental Neuro-psychobiology Laboratory,Department of Clinical and Behavioral Neurology, IRCCS Santa Lucia Foundation, Rome, Italy
54. Department of Neurology and Clinical Neurophysiology, University Hospital of Trondheim, Trondheim, Norway
55. Department of Neuromedicine and Movement Science, Norwegian University of Science and Technology, Trondheim, Norway
56. Dementia Centre for Research Collaboration, School of Psychiatry, University of New South Wales, Sydney, Australia
57. Biosciences, School of Science and Technology, Nottingham Trent University, Nottingham UK
58. Centro de Neuropsiquiatría y Neurología de la Conducta (CENECON), Facultad de Medicina, Universidad de Buenos Aires (UBA), C.A.B.A, Buenos Aires, Argentina.
59. Departamento Ciencias Fisiológicas UAII, Facultad de Medicina, UBA, C.A.B.A, Buenos Aires, Argentina.
60. Hospital Interzonal General de Agudos Eva Perón, San Martín, Buenos Aires, Argentina.
61. Unidad de Trastornos del Movimiento, Servicio de Neurología y Neurofisiología. Instituto de Biomedicina de Sevilla (IBiS), Hospital Universitario Virgen del Rocío/CSIC/Universidad de Sevilla, Seville, Spain
62. Institute for Stroke and Dementia Research, Klinikum der Universität München, Ludwig-Maximilians-Universität LMU, Munich, Germany.
63. German Center for Neurodegenerative Diseases (DZNE, Munich), Munich, Germany.
64. Faculty of Medical & Health Sciences, University of Auckland, New Zealand
65. Wales Centre for Ageing & Dementia Research, Swansea University, Wales, New Zealand
66. UFIEC, Instituto de Salud Carlos III, , Madrid, Spain

67. Inserm, Bordeaux Population Health Research Center, UMR 1219, Univ. Bordeaux, ISPED, CIC 1401-EC, Univ Bordeaux,Bordeaux, France
68. CHU de Bordeaux, Pole santé publique, Bordeaux, France
69. Grupo de Medicina Xenómica, Centro Nacional de Genotipado (CEGEN-PRB3-ISCIII). Universidade de Santiago de Compostela, Santiago de Compostela, Spain.
70. Fundación Pública Galega de Medicina Xenómica- CIBERER-IDIS, University of Santiago de Compostela, Santiago de Compostela, Spain.
71. Institute of Gerontology and Geriatrics, Department of Medicine and Surgery, University of Perugia Perugia, Italy
72. Normandie Univ, UNIROUEN, Inserm U1245 and CHU Rouen, Department of Genetics and CNR-MAJ, Rouen, France
73. Unit of Clinical Pharmacology, University Hospital of Cagliari, Cagliari, Italy
74. Radboudumc Alzheimer Center, Department of Geriatrics, Radboud University Medical Center, Nijmegen, the Netherlands
75. Institute for Regenerative Medicine, University of Zürich, Schlieren, Switzerland
76. Unidad Clínica de Enfermedades Infecciosas y Microbiología. Hospital Universitario de Valme, Sevilla, Spain
77. Department of Neuroscience, Catholic University of Sacred Heart, Fondazione Policlinico Universitario A. Gemelli IRCCS, Rome, Italy
78. University of Bari, “A. Moro”, Bari, Italy
79. School of Medicine, University of Thessaly, Larissa, Greece
80. University Bordeaux, Inserm, Bordeaux Population Health Research Center, France
81. Department of Neurology, University Medical Center Groningen, the Netherlands
82. Department of Neurology, Bordeaux University Hospital, Bordeaux, France
83. Department of Psychiatry, Psychosomatics and Psychotherapy, Center of Mental Health, University Hospital, Wuerzburg
84. UKDRI@ Cardiff, School of Medicine, Cardiff University, Cardiff, UK
85. Munich Cluster for Systems Neurology (SyNergy), Munich, Germany.
86. Technical University of Munich, School of Medicine, Klinikum rechts der Isar, Department of Psychiatry and Psychotherapy, Munich, Germany
87. Institute of Cognitive Neurology and Dementia Research (IKND), Otto-Von-Guericke University, Magdeburg, Germany.
88. German Center for Neurodegenerative Diseases (DZNE), Magdeburg, Germany.
89. Faculty of Medicine, University of Lisbon, Portugal
90. Department of Psychiatry, Social Medicine Center East- Donauspital, Vienna, Austria
91. Institute of Clinical Medicine, University of Oslo, Oslo, Norway.
92. Dementia Research Centre, UCL Queen Square Institute of Neurology, London, United Kingdom
93. Unidad de Demencias, Servicio de Neurología y Neurofisiología. Instituto de Biomedicina de Sevilla (IBiS), Hospital Universitario Virgen del Rocío/CSIC/Universidad de Sevilla, Seville, Spain
94. Instituto de Investigacion Sanitaria ‘Hospital la Paz’ (IdIPaz), Madrid, Spain
95. Centro de Biología Molecular Severo Ochoa (UAM-CSIC), Madrid, Spain
96. Hospital Universitario la Paz, Madrid, Spain
97. Department of geriatric Psychiatry, Central Institute for Mental Health, Mannheim, University of Heidelberg, Germany
98. Alzheimer Research Center & Memory Clinic, Andalusian Institute for Neuroscience, Málaga, Spain.
99. Hospital Universitario Ramon y Cajal, IRYCIS, Madrid
100. Department of Psychiatry and Psychotherapy, Medical University of Vienna, Vienna, Austria
101. CAEBI, Centro Andaluz de Estudios Bioinformáticos, Sevilla, Spain.
102. Karolinska Institutet, Center for Alzheimer Research, Department NVS, Division of Neurogeriatrics, Stockholm, Sweden
103. Aging Research Center, Department of Neurobiology, Care Sciences and Society, Karolinska Institutet and Stockholm University, Stockholm, Sweden
104. Institute of Public Health, University of Cambridge, UK

105. Department of Child and Adolescent Psychiatry and Psychotherapy, University Hospital of Psychiatry Zurich, University of Zurich, Zurich, Switzerland
106. Neuroscience Center Zurich, University of Zurich and ETH Zurich, Switzerland
107. Zurich Center for Integrative Human Physiology, University of Zurich, Switzerland
108. Human Genetics, School of Life Sciences, Life Sciences Building, University Park, University of Nottingham, Nottingham, UK
109. A.I Virtanen Institute for Molecular Sciences, University of Eastern Finland, Kuopio, Finland
110. Department of Neurology, Medical School, University of Cyprus, Cyprus
111. Sorbonne University, GRC n° 21, Alzheimer Precision Medicine Initiative (APMI), AP-HP, Pitié-Salpêtrière Hospital, Boulevard de l'hôpital, Paris, France
112. Eisai Inc., Neurology Business Group, 100 Tice Blvd, Woodcliff Lake, NJ 07677, USA
113. Reta Lila Weston Research Laboratories, Department of Molecular Neuroscience, UCL Institute of Neurology, London, UK.
114. Institute of Human Genetics, University of Bonn, School of Medicine & University Hospital Bonn, Bonn, Germany
115. Insitute of Clinical Medicine - Neurology, University of Eastern, Kuopio, Finland
116. Institute of Clinical Medicine – Internal Medicine, University of Eastern Finland, Kuopio, Finland
117. Clinical and Experimental Science, Faculty of Medicine, University of Southampton, Southampton, UK.
118. Unit for Hereditary dementias, Karolinska University Hospital-Solna, Stockholm, Sweden
119. Division of Clinical Geriatrics, Center for Alzheimer Research, Care Sciences and Society (NVS), Karolinska Institutet, Stockholm, Sweden
120. Institute of Public Health and Clinical Nutrition, University of Eastern Finland, Kuopio, Finland
121. Neuroepidemiology and Ageing Research Unit, School of Public Health, Imperial College London, London, United Kingdom
122. Stockholms Sjukhem, Research & Development Unit, Stockholm, Sweden
123. Department of Psychiatry and Psychotherapy, Universitätsklinikum Erlangen, and Friedrich-Alexander Universität Erlangen-Nürnberg, Erlangen, Germany.
124. Laboratory of Cognitive Neuroscience, School of Psychology, Aristotle University of Thessaloniki, Thessaloniki, Greece
125. Neurology Service, Marqués de Valdecilla University Hospital (University of Cantabria and IDIVAL), Santander, Spain.
126. Stockholm Gerontology Research Center, Stockholm, Sweden
127. Public Health Promotion Unit, Finnish Institute for Health and Welfare, Helsinki, Finland
128. Memory Clinic, Department of Neurology, Charles University, 2nd Faculty of Medicine and Motol University Hospital, Czech Republic
129. International Clinical Research Center, St. Anne’s University Hospital Brno, Brno, Czech Republic
130. Department of Neurology. Hospital Universitario Donostia. OSAKIDETZA-Servicio Vasco de Salud, San Sebastian, Spain
131. School of Health Sciences, Bangor University, UK
132. Unit of Neurology, University of Parma and AOU, Parma, Italy
133. Institute of Neurology, Catholic University of the Sacred Heart , Rome, Itlay
134. MRC Prion Unit at UCL, UCL Institute of Prion Diseases, London, UK
135. Clinic of Neurology, UH "Alexandrovska", Medical University - Sofia, Sofia, Bulgaria
136. Institute for Urban Public Health, University Hospital of University Duisburg-Essen, Essen, Germany
137. Department of Neurology, ErasmusMC, Rotterdam, The Netherlands
138. Neurological Tissue Bank of the Biobanc-Hospital Clinic-IDIBAPS, Institut d'Investigacions Biomèdiques August Pi i Sunyer, Barcelona, Spain.
139. Alzheimer’s disease and other cognitive disorders Unit. Neurology Department, Hospital Clinic , Barcelona, Spain
140. Laboratory of Brain Aging and Neurodegeneration- FIL-CONICET, Buenos Aires, Argentina
141. Human Genetics, School of Life Sciences, University of Nottingham, UK
142. IRCCS Fondazione Don Carlo Gnocchi, Florence, Italy

143. Department of Clinical Biochemistry, Herlev and Gentofte Hospital, Herlev, Denmark
144. Department of Clinical Medicine, University of Copenhagen, Copenhagen, Denmark
145. DIMEC, University of Parma, Parma, Italy
146. Institut de Biomedicina de València-CSIC (valència, Spain) CIBERNED.
147. Unitat Mixta de de Neurología y Genética, Institut d'Investigació Sanitària La Fe (València, Spain)
148. Univ. Lille, CNRS, Inserm, CHU Lille, Institut Pasteur de Lille, US 41-UMS 2014-PLBS, bilille, Lille, France.
149. Institute of Psychiatry and Psychotherapy, Charité-Universitätsmedizin Berlin, Corporate Member of Freie Universität Berlin, Humboldt-Universität Zu Berlin, and Berlin Institute of Health, Berlin, Germany.
150. German Center for Neurodegenerative Diseases (DZNE), Berlin, Germany.
151. Department of Biomedical Sciences, University of Cagliari, Italy
152. CHUV, Old Age Psychiatry, Department of Psychiatry, Lausanne, Switzerland
153. Old Age Psychiatry, Department of Psychiatry, Lausanne University Hospital, Lausanne, Switzerland
154. Department of Geriatric Psychiatry, University Hospital of Psychiatry Zürich, Zürich, Switzerland
155. Department of Neuropsychiatry and Laboratory of Molecular Psychiatry, Charité, Charitéplatz 1, 10117 Berlin, Germany
156. Department of Clinical Biochemistry, Rigshospitalet, Copenhagen, Denmark
157. Maastricht University, Department of Psychiatry & Neuropsychologie, Alzheimer Center Limburg, Maastricht, the Netherlands
158. Depatamento de Especialidades Quirúrgicas, Bioquímica e Inmunología. Facultad de Medicina. Universidad de Málaga. Málaga, Spain
159. Delft Bioinformatics Lab, Delft University of Technology, Delft, The Netherlands
160. Institute of Social Medicine, Occupational Health and Public Health, University of Leipzig, 04103 Leipzig, Germany.
161. Center of Mental Health, Clinic and Policlinic of Psychiatry, Psychosomatics and Psychotherapy, University Hospital of Würzburg, Wuerzburg, Germany
162. Department of Research and Innovation, Helse Fonna, Haugesund Hospital, Haugesund, Norway.
163. The University of Bergen, Institute of Clinical Medicine (K1), Bergen Norway
164. Departamento de Especialidades Quirúrgicas, Bioquímicas e Inmunología, School of Medicine, University of Málaga, Málaga, Spain.
165. Department of Neuroscience and Mental Health, AOU Città della Salute e della Scienza di Torino, Torino, Italy
166. Athens Association of Alzheimer’s disease and Related Disorders, Athens, Greece
167. Department of Geriatrics, St. Olav’s Hospital, Trondheim University Hospital, Norway
168. Department of Immunology, Hospital Universitario Doctor Negrín, Las Palmas de Gran Canaria, Spain.
169. Neurology department-Hospital Clínic, IDIBAPS, Universitat de Barcelona, Barcelona, Spain.
170. Taub Institute for Research in Alzheimer’s Disease and the Aging Brain, The Gertrude H. Sergievsky Center, Depatment of Neurology, Columbia University, New York, NY
171. 1st Department of Neurology, Aiginition Hospital, National and Kapodistrian University of Athens, Medical School, Greece
172. LVR-Hospital Essen, Department of Psychiatry and Psychotherapy, Medical Faculty, University of Duisburg-Essen, Virchowstr. 174, 45147 Essen, Germany
173. Department of Primary Medical Care, University Medical Centre Hamburg-Eppendorf, 20246 Hamburg, Germany.
174. Institute of Medical Biometry, Informatics and Epidemiology, University Hospital of Bonn, Bonn, Germany.
175. Department of Geriatric Medicine, Oslo University Hospital, Oslo, Norway
176. Laboratory for Advanced Hematological Diagnostics, Department of Hematology and Stem Cell Transplant, Lecce, Italy

177. Department of Biomedical Sciences, Section of Neuroscience and Clinical Pharmacology, University of Cagliari, Italy
178. Department of Psychiatry, Namsos Hospital, Namsos, Norway
179. Department of Internal medicine and Biostatistics, ErasmusMC, Rooterdam, The Netherlands
180. Department of Radiology&Nuclear medicine, ErasmusMC, Totterdam, The Netherlands
181. Laboratory for Cognitive Neurology, Department of Neurosciences, University of Leuven, Belgium
182. Neurology Department, University Hospitals Leuven, Leuven, Belgium
183. Department of Psychiatry and Psychotherapy, University Medical Center Goettingen, Goettingen, Germany
184. Department of Psychiatry, Harvard Medical School, McLean Hospital, Belmont, MA, USA
185. Normandie Univ, UNIROUEN, Inserm U1245, CHU Rouen, Department of Neurology and CNR-MAJ, F 76000, Normandy Center for Genomic and Personalized Medicine, Rouen, France
186. German Center for Neurodegenerative Diseases (DZNE), Goettingen, Germany
187. Medical Science Department, iBiMED, Aveiro, Portugal
188. Department of Nutrition and Diatetics, Harokopio University, Athens, Greece
189. Neurosciences Area. Instituto Biodonostia. San Sebastian, Spain
190. Department of Clinical Biochemistry, Hematology and Immunology, Na Homolce Hospital, Prague, Czech republic
191. Institute of Gerontology and Geriatrics, Department of Medicine, University of Perugia Perugia (Italy)
192. Insitute of Biomedicine, University of Eastern Finland, Finland
193. Center for Life Course Health Research, University of Oulu, Oulu, Finland
194. Medical Research Center Oulu, Oulu University Hospital, Oulu, Finland
195. University of Helsinki and Helsinki University Hospital, Helsinki, Finland
196. Division of Psychological Medicine and Clinial Neurosciences, MRC Centre for Neuropsychiatric Genetics and Genomics, Cardiff University, UK
197. Kings College London, Institute of Psychiatry, Psychology and Neuroscience, UK
198. Division of Psychiatry, University College London, UK
199. Institute of Public Health, University of Cambridge, Cambridge, UK
200. Institute of Genetics, Queens Medical Centre, University of Nottingham, Nottingham, UK
201. XXX
202. Ageing Group, Centre for Public Health, School of Medicine, Dentistry and Biomedical Sciences, Queen's University Belfast, UK
203. The Wellcome Trust Sanger Institute, Wellcome Trust Genome Campus, Hinxton, Cambridge, UK.
204. Dementia Research Centre, Department of Neurodegenerative Disease, UCL Institute of Neurology, London, UK
205. Mercer's Institute for Research on Ageing, St James' Hospital, Dublin, Ireland
206. Department of Molecular Neuroscience, UCL, Institute of Neurology, London, UK
207. School of Biotechnology, Dublin City University, Dublin, Ireland
208. Centre for Public Health, School of Medicine, Dentistry and Biomedical Sciences, Queens University, Belfast, UK
209. Department of Psychiatry, University of Oxford, Oxford, UK
210. Department of Basic and Clinical Neuroscience, Institute of Psychiatry, Psychology and Neuroscience, Kings College London, London UK
211. Genetic Epidemiology, QIMR Berghofer Medical Research Institute, Herston, Queensland, Australia
212. Division of Neuroscience and Experimental Psychology, School of Biological Sciences, Faculty of Biology, Medicine and Health, University of Manchester, Manchester Academic Health Science Centre, Manchester M13 9PT, UK
213. Oxford Project to Investigate Memory and Ageing (OPTIMA), University of Oxford, Level 4, John Radcliffe Hospital, Oxford, UK
214. Department of Mental Health Sciences, University College London, London, UK

215. Ageing Group, Centre for Public Health, School of Medicine, Dentistry and Biomedical Sciences, Queen’s University Belfast, Belfast, UK.
216. Dementia Research Centre, UCL, London, UK
217. Servei de Neurologia. Hospital Clínic Universitari de València, Spain
218. Department of Radiology, University Hospital Bonn, Bonn, Germany
219. German Center for Neurodegenerative Diseases (DZNE), Tübingen, Germany
220. Section for Dementia Research, Hertie Institute for Clinical Brain Research and Department of Psychiatry, Tübingen, Germany
221. Department of Psychiatry and Psychotherapy, University Hospital, LMU Munich, Munich, Germany
222. Service of Neurology, University Hospital Marqués de Valdecilla, IDIVAL, University of Cantabria, Santander, Spain
223. Molecular Medicine Center, Department of Medical chemistry and biochemistry, Medical University of Sofia, Bulgaria
224. Department of Psychiatry, Psychosomatics and Psychotherapy, Center of Mental Health, University Hospital of Würzburg, Germany
225. ENYS (Estudio en Neurociencias y Sistemas Complejos) CONICET- Hospital El Cruce "Nestor Kirchner"- UNAJ, Argentina
226. HIGA Eva Perón, Buenes Aires, Agentina
227. Neurología Clinica, Buenes Aires, Agentina
228. Dirección de Atención de Adultos Mayores del Min. Salud Desarrollo Social y Deportes de la Pcia. de Mendoza, Argentina
229. Laboratorio de Genética Forense del Ministerio Público de la Pcia. de La Pampa, Argentina
230. Fundacion Sinapsis, Santa Rosa, Argentina
231. Hospital Dr. Lucio Molas, Santa Rosa; Fundacion Ayuda Enfermo Renal y Alta Complejidad (FERNAC), Santa Rosa, Argentina
232. Laboratory of Brain Aging and Neurodegeneration- FIL, Buneos Aires, Argentina
233. Centro de Neuropsiquiatría y Neurología de la Conducta (CENECON), Facultad de Medicina, Universidad de Buenos Aires (UBA), C.A.B.A, Buenos Aires, Argentina
234. Center for Neurosciences, Vrije Universiteit Brussel (VUB), Brussels, Belgium
235. Reference Center for Biological Markers of Dementia (BIODEM), Institute Born-Bunge, University of Antwerp, Antwerp, Belgium
236. Institute Born-Bunge, University of Antwerp, Antwerp, Belgium
237. Department of Neurology, UZ Brussel, Brussels, Belgium
238. Fondazione IRCCS, Istituto Neurologico Carlo Besta, Milan Italy
239. Department of Clinical Biochemistry, Herlev and Gentofte Hospital, Herlev Denmark
240. Centre for Memory Disturbances, Lab of Clinical Neurochemistry, Section of Neurology, University of Perugia, Italy
241. University of Milan, Milan, Italy
242. Department of Neurology, UMC Utrecht Brain Center, Utrecht, the Netherlands
243. Translational Health Sciences, Bristol Medical School, University of Bristol, Bristol, BS16 1LE, UK
244. Univ Lille Inserm 1172, CHU Clinical and Research Memory Research Centre (CMRR) of Distalz, Licend, Lille France
245. Neurodegenerative Brain Diseases Group, VIB Center for Molecular Neurology, VIB, Antwerp, Belgium
246. Cambridge Institute for Medical Research and UK Dementia Research Institute, University of Cambridge, Cambridge, UK
247. German Center for Neurodegenerative Diseases (DZNE), Rostock, Germany
248. Centro de Investigación Biomédica en Red de Diabetes y Enfermedades Metabólicas Asociadas, CIBERDEM, Spain, Hospital Clínico San Carlos, Madrid, Spain
249. Glenn Biggs Institute for Alzheimer’s and Neurodegenerative Diseases, San Antonio, TX, USA

The GR@ACE study group

Aguilera N[1], Alarcon E[1], Alegret M[1,2], Boada M[1,2], Buendia M[1], Cano A[1], Cañabate P[1,2], Carracedo A[4,5], Corbatón-Anchuelo A[6], de Rojas I[1], Diego S[1], Espinosa A[1,2], Gailhajenet A[1], García-González P[1,2], Guitart M[1], González-Pérez A[7], Ibarria M[1], Lafuente A[1], Macias J[8], Maro~nas O[4], Martín E[1], Martínez MT[6], Marquié M[1,2], Montrreal L[1], Moreno- Grau S[1,2], Moreno M[1], R. Nuñez-Llaves R[1], Olivé C[1] , Orellana A[1], Ortega G[1,2], Pancho A[1], Pelej`a E[1], Pérez-Cordon A[1], Pineda JA[8], Puerta R[1], Preckler S[1], Quintela I[3], Real LM[3,8], Rosende- Roca M[1], Ruiz A[1,2], Sáez ME[7], Sanabria A[1,2], Serrano-Rios M[6], Sotolongo-Grau O[1], Tárraga L[1,2], Valero S[1,2], Vargas L[1].

1. Research Center and Memory clinic. ACE Alzheimer Center Barcelona, Universitat Internacional de Catalunya, Spain.
2. CIBERNED, Center for Networked Biomedical Research on Neurodegenerative Diseases, National Institute of Health Carlos III, Ministry of Economy and Competitiveness, Spain,
3. Dep. of Surgery, Biochemistry and Molecular Biology, School of Medicine. University of Malaga. Malaga, Spain,
4. Grupo de Medicina Xenómica, Centro Nacional de Genotipado (CEGEN-PRB3-ISCIII). Universidad de Santiago de Compostela, Santiago de Compostela, Spain.
5. Fundación Pública Galega de Medicina Xenómica- CIBERER-IDIS, Santiago de Compostela, Spain.
6. Centro de Investigación Biomédica en Red de Diabetes y Enfermedades Metabólicas Asociadas, CIBERDEM, Spain, Hospital Clínico San Carlos, Madrid, Spain,
7. CAEBI. Centro Andaluz de Estudios Bioinformáticos, Sevilla, Spain
8. Unidad Clínica de Enfermedades Infecciosas y Microbiología. Hospital Universitario de Valme, Sevilla, Spain.

DEGESCO consortium

Adarmes-Gómez AD[1,2], Alarcón-Martín E[3], Alonso MD[4], Álvarez I[5], Álvarez V[6,7], Amer-Ferrer G[8], Antequera M[9], Antúnez C[9], Baquero M[10], Bernal M[11], Blesa R[2,12], Boada M[2,3], Buiza-Rueda D[1,2], Bullido MJ[2,14,15], Burguera JA[10], Calero M[2,16,17], Carrillo F[1,2], Carrión-Claro M[1,2], Casajeros MJ[18], Clarimón J[2,12], Cruz-Gamero JM[13], de Pancorbo MM[19], de Rojas I[2,3], del Ser T[15], Diez-Fairen M[5], Escuela R[1,2], Garrote-Espina L[1,2], Fortea J[2,12], Franco E[11], Frank-García A[2,15,20], García-Alberca JM[21], Garcia Madrona S[17], Garcia-Ribas G[17], Gómez-Garre P[1,2], Hevilla S[21], Jesús S[1,2], Labrador Espinosa MA[1,2], Lage C[2,22], Legaz A[9], Lleó A[2,12], López de Munáin A[23], López-García S[2,22], Macias-García D[1,2], Manzanares S[8,24], Marín M[11], Marín-Muñoz J[9], Marín T[21], Marquié M[2,3], Martín Montes A[2,14,20], Martínez B[9], Martínez C[7,25], Martínez V[9], Martínez-Lage Álvarez P[26], Medina M[2,15], Mendioroz Iriarte M[27], Menéndez- González M[7,28], Mir P[1,2], Montrreal L[3], Orellana A[3], Pastor P[5], Pérez Tur J[2,29,30], Periñán-Tocino T[1,2], Pineda-Sánchez R[1,2], Piñol Ripoll G[2,31], Rábano A[2,16,32], Real de Asúa D[33], Rodrigo S[11], Rodríguez-Rodríguez E[2,22], Royo JL[13], Ruiz A[2,3], Sanchez del Valle Díaz R[34], Sánchez-Juan P[16], Sastre I[2,14], Sotolongo-Grau O[3], Valero S[2,3], Vicente MP[9], Vigo-Ortega R[1,2], Vivancos L[9].

1. Unidad de Trastornos del Movimiento, Servicio de Neurología y Neurofisiología. Instituto de Biomedicina de Sevilla (IBiS), Hospital Universitario Virgen del Rocío/CSIC/Universidad de Sevilla, Seville, Spain,
2. CIBERNED, Network Center for Biomedical Research in Neurodegenerative Diseases, National Institute of Health Carlos III, Spain,
3. Research Center and Memory clinic. ACE Alzheimer Center Barcelona, Universitat Internacional de Catalunya, Spain,

4. Servei de Neurologia. Hospital Clínic Universitari de València, Spain.
5. Fundació per la Recerca Biom`edica i Social Mútua Terrassa, and Memory Disorders Unit, Department of Neurology, Hospital Universitari Mutua de Terrassa, University of Barcelona School of Medicine, Terrassa, Barcelona, Spain,
6. Laboratorio de Genética Hospital Universitario Central de Asturias, Oviedo, Spain
7. Instituto de Investigación Biosanitaria del Principado de Asturias (ISPA), Oviedo, Spain
8. Department of Neurology, Hospital Universitario Son Espases, Palma, Spain,
9. Unidad de Demencias. Hospital Clínico Universitario Virgen de la Arrixaca, Palma, Spain,
10. Servei de Neurologia, Hospital Universitari i Polit`ecnic La Fe, Velencia, Spain
11. Unidad de Demencias, Servicio de Neurología y Neurofisiología. Instituto de Biomedicina de Sevilla (IBiS), Hospital Universitario Virgen del Rocío/CSIC/Universidad de Sevilla, Seville, Spain
12. Memory Unit, Neurology Department and Sant Pau Biomedical Research Institute, Hospital de la Santa Creu i Sant Pau, Universitat Aut`onoma de Barce- lona, Barcelona, Spain,
13. Dep. of Surgery, Biochemistry and Molecular Biology, School of Medicine. University of Málaga. Málaga, Spain
14. Centro de Biologia Molecular Severo Ochoa (C.S.I.C.-U.A.M.), Universidad Autonoma de Madrid, Madrid, Spain
15. Instituto de Investigacion Sanitaria 'Hospital la Paz' (IdIPaz), Madrid, Spain,
16. CIEN Foundation, Queen Sofia Foundation Alzheimer Center, Madrid, Spain
17. Instituto de Salud Carlos III (IS- CIII), Madrid, Spain;
18. [18]Hospital Universitario Ramón y Cajal; Madrid, Spain,
19. BIOMICs, País Vasco; Centro de Investigación Lascaray. Universidad del País Vasco UPV/EHU, Vitoria-Gasteiz, Spain
20. Neurology Service, Hospital Universitario La Paz (UAM), Madrid, Spain,
21. Alzheimer Research Center & Memory Clinic. Andalusian Institute for Neuroscience. Málaga, Spain,
22. Neurology Service, Marqués de Valdecilla University Hospital (University of Cantabria and IDIVAL), Santander, Spain,
23. Hospital Donostia de San Sebastían, San Sebastían, Spain
24. Fundación para la Formación e Investigación Sanitarias de la Región de Murcia, Palma Spain
25. Servicio de Neurología -Hospital de Cabue~nes-Gijón, Gijón, Spain
26. Centro de Investigacio´n y Terapias Avanzadas. Fundación CITA-alzheimer, San Sebastian, Spain
27. Navarrabiomed, Pamplona, Spain
28. Servicio de Neurología Hospital Universitario Central de Asturias, Oviedo, Spain
29. Unitat de Gen`etica Molecular. Institut de Biomedicina de Val`encia-CSIC, Vencia, Spain
30. Unidad Mixta de Neurologia Gen`etica. Instituto de Investigación Sanitaria La Fe, Valencia, Spain
31. Unitat Trastorns Cognitius, Hospital Universitari Santa Maria de Lleida, Institut de Recerca Biomédica de Lleida (IRBLLeida), Lleida, Spain
32. BT-CIEN,
33. Hospital Universitario La Princesa, Madrid, Spain,
34. Hospital Clínic Barcelona, Spain

Demgene

Alexey A Shadrin[1,2], Shahram Bahrami[1,2], Arvid Rongve[3,4], Geir Bråthen[5,6], Ingunn Bosnes[7,8], Eystein Stordal[7,8], Lavinia Athanasiu[1,2], Per Selnes[9], Ingvild Saltvedt[5,10], Sigrid B. Sando[5,6], Sverre Bergh[11], Ingun Ulstein[12], Srdjan Djurovic[13,14], Tormod Fladby[9,15], Dag Aarsland[16,17], Geir Selbæk[12,15,18], Ole A. Andreassen[1,2]

1. NORMENT Centre, University of Oslo, Oslo, Norway.
2. Division of Mental Health and Addiction, Oslo University Hospital, Oslo, Norway.

3. Department of Research and Innovation, Helse Fonna, Haugesund Hospital, Haugesund, Norway.
4. The University of Bergen, Institute of Clinical Medicine (K1), Bergen Norway.
5. Department of Neuromedicine and Movement Science, Norwegian University of Science and Technology, Trondheim, Norway.
6. Department of Neurology and Clinical Neurophysiology, University Hospital of Trondheim, Norway.
7. Department of Mental Health, Faculty of Medicine and Health Sciences, Norwegian University of Science and Technology, Trondheim, Norway.
8. Department of Psychiatry, Hospital Namsos, Nord-Trøndelag Health Trust, Namsos, Norway.
9. Department of Neurology, Akershus University Hospital, Lørenskog, Norway.
10 Department of Geriatrics, St. Olav's Hospital, Trondheim University Hospital, Norway.
11. Centre for Old Age Psychiatry Research, Innlandet Hospital Trust, Ottestad, Norway.
12. Department of Geriatric Medicine, Oslo University Hospital, Oslo, Norway.
13. Department of Medical Genetics, Oslo University Hospital, Oslo, Norway.
14. NORMENT, Department of Clinical Science, University of Bergen, Bergen, Norway.
15 Institute of Clinical Medicine, University of Oslo, Oslo, Norway
16. Centre of Age-Related Medicine, Stavanger University Hospital, Norway.
17. Institute of Psychiatry, Psychology & Neuroscience, PO 70, 16 De Crespigny Park, London, SE58AF.
18. Norwegian National Advisory Unit on Ageing and Health, Vestfold Hospital Trust, Tønsberg, Norway.

EADI

Céline Bellenguez[1], Benjamin Grenier-Boley[1], Jacques Epelbaum[2], David Wallon[3], Didier Hannequin[3], Florence Pasquier[4], Claudine Berr[5], Jean-Francois Dartigues[6], Dominique campion[7], Christophe Tzourio[8], Vincent Dermecourt[4], Nathalie Fievet[1], Olivier Hanon[9], Carole Dufouil[8], Alexis Brice[10], Bruno Dubois[11], Karen Ritchie[5], Phillippe Amouyel[1], Jean-Charles Lambert[1]

1. Univ. Lille, Inserm, CHU Lille, Institut Pasteur Lille, U1167-RID-AGE - Facteurs de risque et déterminants moléculaires des maladies liées au vieillissement, F-59000 Lille, France
2. UMR 894, Center for Psychiatry and Neuroscience, INSERM, Université Paris Descartes, F-75000 Paris , France
3. Normandie Univ, UNIROUEN, Inserm U1245, CHU Rouen, Department of Neurology and CNR-MAJ, F 76000, Normandy Center for Genomic and Personalized Medicine, Rouen, France
4. Univ. Lille, Inserm, CHU Lille, UMR1172, Resources and Research Memory Center (MRRC) of Distalz, Licend, Lille France
5. Univ. Montpellier, Inserm U1061, Neuropsychiatry: epidemiological and clinical research, PSNREC, Montpellier, France
6. University Bordeaux, Inserm, Bordeaux Population Health Research Center, France
7. Normandie Univ, UNIROUEN, Inserm U1245 and CHU Rouen, Department of Genetics and CNR-MAJ, Rouen, France
8. University Bordeaux, Inserm, Bordeaux Population Health Research Center, France
9. Université de Paris, EA 4468, APHP, Hôpital Broca, Paris, France
10. Inserm U1127, CNRS UMR7225, Sorbonne Universités, UPMC Univ Paris 06, UMR_S1127, Institut du Cerveau et de la Moelle épinière, F-75013, Paris, France; 22. APHP, Department of genetics, Pitié-Salpêtrière Hospital, 75013, Paris, France
11. Institut de la Mémoire et de la Maladie d'Alzheimer (IM2A), Département de Neurologie, Hôpital de la Pitié-Salpêtrière, AP-HP, Paris, France; Institut des Neurosciences Translationnelles de

Paris (IHU-A-ICM), Institut du Cerveau et de la Moelle Epinière (ICM), Paris, France; 26. INSERM, CNRS, UMR-S975, Institut du Cerveau et de la Moelle Epinière (ICM), Paris, France; Sorbonne Universités, Université Pierre et Marie Curie, Hôpital de la Pitié-Salpêtrière, AP-HP, Paris, France

GERAD

Denise Harold[1], Paul Hollingworth[2], Rebecca Sims[2], Amy Gerrish[2], Nicola Denning[2], Amy Williams[2], Charlene Thomas[2], Alun Meggy[2,3], Rachel Marshall[2], Chloe Davies[2], Lauren Luckcuck[2,3], William Nash[2], Kimberley Dowzell[2], Atahualpa Castillo Morales[2,3], Mateus Bernardo-Harrington[2,3], Patrick Kehoe[4], Per Hoffmann[4], Seth Love[4], James Turton[5], Jenny Lord[5], Kristelle Brown[5], Kevin Morgan[5], Emma Vardy[6], Elizabeth Fisher[7], Jason D. Warren[7], Jonathan M. Schott[7], Martin Rossor[7], Natalie S. Ryan[7], Nick C. Fox[7], Rita Guerreiro[7], Simon Mead[7], James Uphill[8], John Collinge[8], Michelle Lupton[8], Ammar Al-Chalabi[9], Christopher E. Shaw[9], Nick Bass[10], Richard Abraham[11], Reinhard Heun[11], Heike Kölsch[11], Britta Schürmann[11], Frank Jessen[11,17], Wolfgang Maier[11,17],André Lacour[12], Christine Herold[12], Simon Lovestone[13], Bernadette McGuinness[14], David Craig[14], Janet A. Johnston[14], Michael Gill[14], Peter Passmore[14], Stephen Todd[14], John Powell[15], Petra Proitsi[15], Yogen Patel[15], Angela Hodges[16], Tim Becker[17,19], A. David Smith[20], Donald Warden[20], Gordon Wilcock[20], Robert Clarke[21], Aoibhinn Lynch[22], Brian Lawlor[22], Michael Gill[22, 23], Andrew McQuillin[24], Gill Livingston[24], John Hardy[25], David C. Rubinsztein[26], Carol Brayne[27], Rhian Gwilliam[28], Panagiotis Deloukas[28], Yoav Ben-Shlomo[29], David Mann[30], Nigel M. Hooper[31], Stuart Pickering-Brown[31], Clive Holmes[32], Rebecca Sussams[32], Nick Warner[33], Anthony Bayer[34], Andrew B. Singleton[35], Annette M Hartmann[36], Dan Rujescu[36], Ina Giegling[36], Harald Hampel[37, 38], Martin Dichgans[39], Isabella Heuser[40], Dmitriy Drichel[41], Norman Klopp[42], Markus M. Nöthen[43, 44], Manuel Mayhaus[45], Matthias Riemenschneider[45], Sabrina Pinchler[45], Thomas Feulner[45], Wei Gu[45], Hendrik van den Bussche[46], Martin Scherer[46], Jens Wiltfang[47], Johannes Kornhuber[48], Michael Hüll[49], Lutz Frölich[50], H-Erich Wichmann[51], Karl-Heinz Jöckel[52], Susanne Moebus[52], Steffi Riedel-Heller[53], John Kauwe[54], John Morris[55,58], Kevin Mayo[55,56,57], Magda Tsolaki[59], Michael O'Donovan[2], Lesley Jones[2], Michael Owen[2], Valentina Escott-Price[2], Alfredo Ramirez[18, 19], Peter Holmans[2], Julie Williams[2,3]

1. School of Biotechnology, Dublin City University, Dublin, Ireland.
2. Division of Psychological Medicine and Clinical Neurosciences, Medical Research Council (MRC) Centre for Neuropsychiatric Genetics & Genomics, Cardiff University, Cardiff, UK.
3. UK Dementia Research Institute at Cardiff, Cardiff University, Cardiff, UK.
4. University of Bristol Medical School, Learning & Research level 2, Southmead Hospital, Bristol, UK.
5. Institute of Genetics, Queen's Medical Centre, University of Nottingham, UK
6. Institute for Ageing and Health, Newcastle University, Biomedical Research Building, Campus for Ageing and Vitality, Newcastle upon Tyne, UK
7. Department of Neurodegenerative Disease, UCL Institute of Neurology, London, UK.
8. Department of Neurodegenerative Disease, MRC Prion Unit at UCL, Institute of Prion Diseases, London, UK
9. MRC Centre for Neurodegeneration Research, Department of Clinical Neuroscience, King's College London, Institute of Psychiatry, London, UK.
10. Division of Psychiatry, University College London, London, UK.
11. Department of Psychiatry and Psychotherapy, University of Bonn, Bonn, Germany
12. Deutsches Zentrum für Neurodegenerative Erkrankungen (DZNE, Bonn), Bonn, Germany

13. Department of Psychiatry, University of Oxford, Oxford, UK.
14. Ageing Group, Centre for Public Health, School of Medicine, Dentistry and Biomedical Sciences, Queen's University, Belfast, UK.
15. Department of Basic and Clinical Neuroscience, Institute of Psychiatry, Psychology and Neuroscience, King's College London, London, UK.
16. Department of Old Age Psychiatry, Institute of Psychiatry, Psychology and Neuroscience, King's College London, London, UK.
17. German Centre for Neurodegenerative Diseases, Bonn, Germany.
18. Department for Neurodegenerative Diseases and Geriatric Psychiatry, University Hospital Bonn, Bonn, Germany.
19. Institute for Medical Biometry, Informatics and Epidemiology, University of Bonn, Bonn, Germany
20. Oxford Project to Investigate Memory and Ageing (OPTIMA), University of Oxford, Nuffield Department of Clinical Neurosciences, John Radcliffe Hospital, Oxford, UK
21. Oxford Healthy Aging Project, Clinical Trial Service Unit, University of Oxford, Oxford, UK.
22. Mercer's Institute for Research on Aging, St. James's Hospital and Trinity College, Dublin, Ireland.
23. St. James's Hospital and Trinity College, Dublin, Ireland.
24. Department of Mental Health Sciences, University College London, UK.
25. Department of Molecular Neuroscience, UCL, Institute of Neurology, London, UK.
26. Cambridge Institute for Medical Research, University of Cambridge, Cambridge, UK
27. Institute of Public Health, University of Cambridge, Cambridge, UK.
28. The Wellcome Trust Sanger Institute, Hinxton, Cambridge, UK.
29. Population Health Sciences, Bristol Medical School, University of Bristol, Bristol, UK.
30. Clinical Neuroscience Research Group, Greater Manchester Neurosciences Centre, University of Manchester, Salford, UK
31. Division of Neuroscience and Experimental Psychology, School of Biological Sciences, Faculty of Biology, Medicine and Health, University of Manchester, Manchester Academic Health Science Centre, Manchester, UK.
32. Division of Clinical Neurosciences, School of Medicine, University of Southampton, Southampton, UK.
33. Somerset Partnership NHS Trust, Somerset, UK.
34. Institute of Primary Care and Public Health, Cardiff University, University Hospital of Wales, Cardiff, UK.
35. Laboratory of Neurogenetics, National Institute on Aging, National Institutes of Health, Bethesda, MD, 20892, USA.
36. Department of Psychiatry, Martin Luther University Halle-Wittenberg, Halle, Germany.
37. Department of Psychiatry, University of Frankfurt, Frankfurt am Main, Germany.
38. Department of Psychiatry, Ludwig Maximilians University, Munich, Germany.
39. Institute for Stroke and Dementia Research, Klinikum der Universität München, Munich, Germany.
40. Department of Psychiatry and Psychotherapy, Charité University Medicine, Berlin, Germany.
41. Cologne Center for Genomics, University of Cologne, Cologne, Germany.
42. Institute of Epidemiology, Helmholtz Zentrum München, German Research Center for Environmental Health, Neuherberg, Munich, Germany.
43. Institute of Human Genetics, University of Bonn, Bonn, Germany.
44. Department of Genomics, Life & Brain Center, University of Bonn, Bonn, Germany.
45. Department of Psychiatry and Psychotherapy, University Hospital, Saarland, Germany.
46. Institute of Primary Medical Care, University Medical Center Hamburg-Eppendorf, Germany
47. Department of Psychiatry and Psychotherapy, University Medical Center Goettingen, Goettingen, Germany
48. Department of Psychiatry and Psychotherapy, University of Erlangen-Nuremberg, Erlangen, Germany
49. Department of Psychiatry, University of Freiburg, Freiburg, Germany.

50. Central Institute of Mental Health, Medical Faculty Mannheim, University of Heidelberg, Heidelberg, Germany.
51. Institute of Epidemiology, Helmholtz Zentrum München, German Research Center for Environmental Health, Neuherberg, Germany
52. Institute for Medical Informatics, Biometry and Epidemiology, University Hospital of Essen, University Duisburg-Essen, Essen, Germany.
53. Institute of Social Medicine, Occupational Health and Public Health, University of Leipzig, Leipzig, Germany.
54. Departments of Biology, Brigham Young University, Provo, UT, USA.
55. Department of Psychiatry, Washington University School of Medicine, St. Louis, MO, USA.
56. Department of Neurology, Washington University, St. Louis, MO, USA.
57. Department of Genetics, Washington University, St. Louis, MO, USA
58. Hope Center Program on Protein Aggregation and Neurodegeneration, Washington University School of Medicine, St. Louis, MO, USA.
59. Department of Neurology, Medical School, Aristotle University of Thessaloniki, Thessaloniki, Greece.
